# Genetic Characterization of Resistance to *Pyrenophora teres* f. *teres* in the International Barley Differential Canadian Lake Shore

**DOI:** 10.3389/fpls.2019.00326

**Published:** 2019-03-25

**Authors:** Eric Dinglasan, Lee Hickey, Laura Ziems, Ryan Fowler, Anna Anisimova, Olga Baranova, Nina Lashina, Olga Afanasenko

**Affiliations:** ^1^Queensland Alliance for Agriculture and Food Innovation, The University of Queensland, St Lucia, QLD, Australia; ^2^All-Russian Institute of Plant Protection, Saint Petersburg, Russia

**Keywords:** net form net blotch, barley, QTL, genetic resistance, marker-assisted selection

## Abstract

Genetic resistance to net form of net blotch in the international barley differential Canadian Lake Shore (CLS) was characterized and mapped. A doubled haploid (DH) population generated from a cross between CLS and susceptible cultivar Harrington was evaluated at the seedling stage using eight diverse *Pyrenophora teres* f. *teres* (*Ptt*) isolates and at the adult stage in the field using natural inoculum. To effectively map the CLS resistance, comparative marker frequency analysis (MFA) was performed using 8,762 polymorphic DArT-seq markers, where ‘resistant’ and ‘susceptible’ groups each comprised 40 DH lines displaying the most extreme phenotypes. Five DArTseq markers were consistently detected in eight disease assays, which was designated *qPttCLS* and deemed to harbor the locus underpinning CLS resistance. Four of these markers were present onto the barley DArTseq physical map and spans a region between 398203862 and 435526243 bp which were found to consist several genes involved in important plant functions such as disease response and signaling pathways. While MFA only detected the 3H region, genetic analyses based on segregation patterns were inconsistent, suggesting complex inheritance or variation in phenotypic expression of *qPttCLS*, particularly in the field. This study represents progress toward connecting *Ptt* pathotype surveys with the corresponding resistance genes in barley differentials. The markers associated with *qPttCLS* are useful for marker-assisted selection in breeding programs.

## Introduction

Net form of net blotch (NFNB) caused by ascomycete fungus *Pyrenophora teres* f. *teres* (*Ptt*) is considered one of the most widespread and destructive diseases of barley (*Hordeum vulgare* L.) crops worldwide. For susceptible cultivars, NFNB can result in yield losses up to 40% (Steffenson et al., 1991; Murray and Brennan, 2010), and under extreme epidemic conditions may cause even higher losses, up to 70% (Wallwork et al., 2016). Still, the most effective means of control is the use of resistant cultivars.

Resistance of barley to *Ptt* has been documented as both qualitative and quantitative resistance (Lai et al., 2007), suggesting a gene-for-gene interaction and complex genetic interactions, respectively. In both cases, resistance is attributed to the isolate of the pathogen.

*Ptt* is a highly diverse pathogen (Khan and Tekauz, 1982; Steffenson and Webster, 1992; Robinson and Jalli, 1997; Afanasenko, 2001; Serenius, 2006; Liu et al., 2012). However, the measure of virulence diversity is limited by the number of differential genotypes used in the tests (Wallwork et al., 2016). The barley cultivar, Canadian Lake Shore (CLS), has been used in numerous studies examining the virulence of local *Ptt* isolates in different barley growing regions around the world (Gray, 1966; Gacek, 1985; Steffenson, 1988; Steffenson and Webster, 1992; Afanasenko et al., 1995; Minarikova, 1995; Minarikova and Polisenska, 1999; Gupta and Loughman, 2001). Based on results from these studies, CLS is a good genotype for discriminating *Ptt* isolates. For this reason, CLS was included in the international set of barley differentials – established to standardize the characterization of *Ptt* populations globally (Afanasenko et al., 2009). However, knowledge of the genetic control(s) of resistance in CLS is unknown. This insight is essential to connect outcomes from pathogen virulence studies to genomic regions conferring resistance and/or susceptibility in the host. Such information will empower breeders to assemble effective host resistance in new barley cultivars.

In this study we performed genetic characterization of CLS resistance at both seedling and adult growth stages using diverse isolates sourced from Russia, Belarus, Germany, Canada, and South Africa. We examined a doubled haploid (DH) population derived from a cross between CLS and susceptible cultivar Harrington. Marker frequency analysis (MFA) was performed based on lines representing ‘resistant’ and ‘susceptible’ classes, in order to effectively map the CLS resistance and identify DNA markers useful for marker-assisted selection (MAS).

## Materials and Methods

### Plant Materials

A total of 101 anther-culture-derived DH lines were developed from F1 plants of the cross between spring barley cultivars CLS and Harrington at the All Russian Research Institute for Plant Protection, Saint Petersburg, Russia. Anthers were cultured according to the method established by Manniner (1997). Notably, both of these cultivars are included in the international set of barley differentials: CLS is a resistant cultivar with good differential ability and Harrington is a susceptible check (Table 1) (Afanasenko et al., 2009).

**Table 1:** Details for the *Pyrenophora teres* f. *teres* isolates used in this study.

Isolate	Geographic	Cultivar^∗^	Year	Virulent/avirulent
	origin		collected	
Len7	Leningrad Region of Russia	Inari	2011	Harrington, Skiff, k-20019, CI5791, k-8755/CLS, Harbin
Ps31	Pskov Region of Russia	Suzdalets	2014	Harrington/CLS, Harbin
Pr11	Primorie, Far East of Russia	Tikhookeanskiy	2014	Harrington/CLS, Skiff, Prior, k-20019, Harbin, CI9825, CI5791, k-8755
Bel1	Minsk Region of Belarus	Gonar	2014	Harrington, Skiff, k-20019/CLS, Prior, Harbin, CI9825, CI5791, k-8755
Vol13	Leningrad Region of Russia	Suzdalets	2014	Harrington, Skiff, Prior, k-20019/CLS, Harbin, CI9825, CI5791, 8755
G5	Germany	NA	2015	Harrington, k-8755, CI9825, Skiff, Prior/CLS, Harbin, CI5791, k-20019
Can11	Canada	Harrington	2012	Harrington, CI9825, Skiff, Prior/k-8755, CLS, Harbin, CI5791, k-20019
SA7	South Africa	NA	2017	Harrington, CI9825, Skiff, Prior/k-8755, CLS, Harbin, CI5791, k-20019

*^∗^Cultivar on which the isolate was collected; NA – cultivar information not available.*

### Pathogen Materials

Eight *Ptt* isolates sourced from different origins were used for screening the DH population and parents in this study (Table 1). Isolates were obtained from infected barley leaves collected between 2011 and 2017. Leaves were surface sterilized with 3% CuSO_4_ for 1 min and then double rinsed in sterile distilled water. Isolates were propagated on Czapek’s modified medium containing the following: 0.5 g/L KH_2_PO_4_, 0.5 g/L MgSO_4_, 0.5 g/L KCl, 1.2 g/L urea, 20 g/L lactose, and 20 g/L agar. The Petri dishes were incubated for 10 days at 20 ± 2°C under constant illumination with a daylight lamp (3000 lux). Single conidia were transferred to the same medium and incubated at the same conditions for 10–12 days. Based on preliminary screening, all isolates were avirulent to CLS.

### Fungal Preparation and Inoculation of Seedlings

The cultures were flooded with distilled water containing 0.01% TWEEN^®^ 20 and conidia were dislodged with a sterile spatula. The spore suspension was filtered through gauze. The spore concentration of the suspension was determined by means of a hemocytometer and adjusted to 5,000 conidia per ml for inoculation.

Evaluation of resistance of DH lines was assayed using the detached leaf method (Afanasenko et al., 1995). Barley seedlings were grown on cotton soaked in water in enameled trays for 8–10 days at 20–22°C with alternating 12 h periods of light (exposure 3000 lux) and dark. Primary leaves were excised and 4–5 cm long segments and were placed in enamel trays (27 cm × 33 cm) on filter paper moistened with sterile water containing 0.004% benzimidazole. For each DH line, leaf segments from two seedlings in four repetitions (total eight seedlings) were placed on the filter paper in different trays. Resistant and susceptible parents were placed in each tray. Inoculation was performed by spraying suspension at a rate of 1 ml per 20 leaf segments. The trays were covered with glass plates and returned to the same light and temperature conditions as used for growing the seedlings.

### Scoring Seedling Infection Response

Five days post-inoculation, the infection response (IR) was recorded using the 10-point scale of Tekauz (1985). For analysis of segregation patterns, lines displaying an average IR of ≤4.9 were considered resistant, and ≥5 were considered susceptible.

### Adult Plant Screening in the Field

The DH lines and two parents Harrington and CLS were screened in 2015 at the adult plant stage in the field, located at the State Cultivars Screening Nursery “Volosovo,” Leningrad Region, Russia. *Ptt* is endemic at this location and net blotch epidemics are observed each year. Lines were planted in 1 m rows (15–20 seeds) in a randomized block design with two replications per line. Parents were planted at the beginning and end of every 10 rows. In order to promote disease development two rows of the highly susceptible cultivar Carlsberg were planted around the experimental plots. No artificial inoculum and no herbicides were applied. NFNB reaction was scored at the growth stage of GS75 (6^th^ to 8^th^ August 2015) using a 1–9 scale, where 1 = very resistant, and 9 = very susceptible.

### Genotyping and Comparative Marker Frequency Analysis

Genomic DNA was extracted for a subset of 94 DH lines and the two parents using the protocol recommended by Diversity Arrays Technology Pty Ltd. (DArT^1^). The samples were genotyped using the Barley GBS 1.0 platform, which returned 8,762 polymorphic silico DArTseq markers.

Marker data was subjected to a quantitative allele frequency analysis technique, known as comparative MFA (Ziems et al., 2017), to identify quantitative trait loci (QTL) associated with *Ptt* resistance. The frequency of alleles contributing resistance (R) contributed by CLS was compared with the frequency of alleles contributing susceptibility (S) by Harrington in the segregating DH progeny. A discriminant value reflecting the difference in allele frequency between the two classes was obtained for each marker according to Wenzl et al. (2006, 2007). This approach can effectively identify genetic loci influencing a trait of interest without the need to generate a linkage map (Wenzl et al., 2007). Each phenotypic class (R or S) comprised 40 DH lines that displayed the most extreme phenotypes in each disease assay. Each marker was subjected to a simple Chi-squared test to detect significant discrimination between the expected and observed allele frequencies. A differential threshold of >0.4 discriminant value and *P* < 0.001 for a marker to be considered associated was determined, ensuring there is a 0.1% probability of detecting an allele frequency difference by chance.

The genomic intervals containing associated DArT-seq markers were displayed on the barley DArTseq consensus map and positioned onto the barley DArTseq physical map using *Pretzel* (Keeble-Gagnère et al., 2019).

The genomic interval of interest harboring the locus associated with *Ptt* response was searched for the presence of genes through *EnsemblPlants* database using the barley genome assembly *Hordeum vulgare* (IBSC_v2) of the International Barley Genome Sequencing Consortium^2^.

## Results

### Seedling Response to *Ptt* Isolates

**FIGURE 1:**
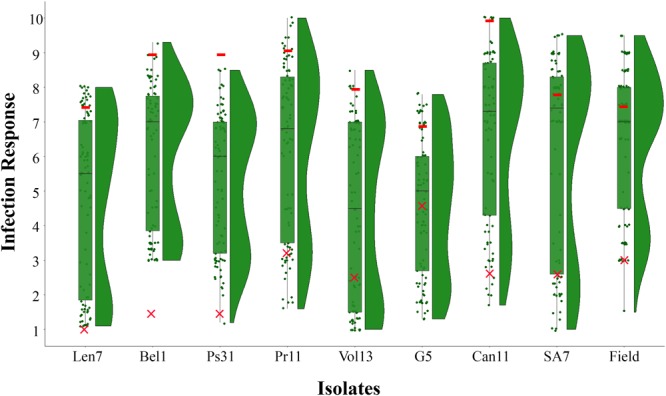
Frequency distribution of infection responses (IRs) in the CLS/Harrington DH population evaluated using eight *Pyrenophora teres* f. *teres* isolates at the seedling stage (Len7, Bel1, Ps31, Pr11, Vol13, G5, Can11, and SA7) and natural inoculum at the adult stage in the field. Box plots show upper and lower quartile where horizontal line represents median IR and overlaid is the raw data points; split violin plots represent the density estimates related to the distribution of CLS/Harrington DH population in each assay. *Symbols* colored in red indicate mean IR displayed by parental genotypes (– Harrington; ×– CLS) in each assay.

**FIGURE 2:**
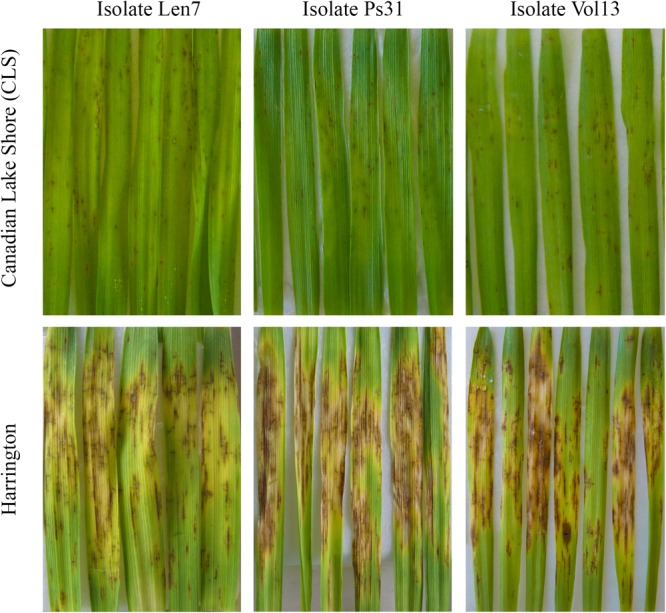
Infection response for parental lines (Canadian Lake Shore and Harrington) for representative isolates of *Pyrenophora teres* f. *teres* (i.e., Len7, Ps31, and Vol13). Leaves from seedlings are displayed.

The resistant parent CLS displayed a low IR across all disease assays (ranging 1.0–4.4), and susceptible parent Harrington displayed a high IR in all assays (ranging 7.0–10.0; Figure 1, 2, Table 2 and Supplementary Table 1). The segregation ratio for four of the eight isolates (i.e., Len7, Ps31, Vol13, and G5) appeared consistent with a single gene inheritance model (i.e., 1:1, Table 2). However, Mendelian analyses based on division of the progeny to susceptible and resistant classes could not explain the segregation pattern observed for isolates Bel1,Pr11, Can11, and SA7 (Table 2).

**Table 2:** The infection response (IR) of doubled haploid (DH) progeny and parents to different *Pyrenophora teres* f. *teres* isolates.

Isolate	IR of DH lines		IR of parents				Genetic	Chi	*P*-value
					Res	Sus	ratio	square	
	Minimum	Maximum	CLS	Harrington					
Len7	1.1	8.0	1.0	7.0	41	57	1:1	2.61	0.106
Bel1	3.0	9.3	1.5	9.0	32	66	1:1	11.80	0.001
Ps31	1.2	8.5	1.5	9.0	41	57	1:1	2.61	0.106
Pr11	1.6	10.0	2.4	8.0	33	63	1:1	9.38	0.002
Vol13	1.0	8.5	3.2	8.9	47	49	1:1	0.04	0.838
G5	1.3	7.8	4.4	7.0	47	42	1:1	0.28	0.596
Can11	1.7	10.0	2.5	10.0	25	61	1:1	15.07	0.000
SA7	1.0	9.5	2.3	7.8	28	54	1:1	8.24	0.004
Field^∗^	1.5	9.5	3.0	7.5	27	68	1:3	0.59	0.441

*The results from Chi-squared (χ^*2*^) analyses are also presented.*

*^∗^Natural inoculum evaluated at the adult growth stage.*

*Res = resistant; Sus = susceptible.*

*P 5% = 3.84 at 1 df.*

The correlation between IRs observed for different *Ptt* isolates in the DH progeny varied from 0.26 to 0.78 (Table 3). The highest degree of correspondence (*r* = 0.78) was found between IR to isolates from Far East of Russia (Pr11) and Pskov Region (Ps31) and the lowest correspondence observed (*r* = 0.26) for IR to isolates from Belarus (Bel1) and Germany (G5) (Table 3).

**Table 3:** Correlation (r) between infection responses observed for different *Pyrenophora teres* f. *teres* isolates in the CLS/Harrington doubled haploid population.

Isolates	Bel1	Ps31	Pr11	Vol13	G5	Can11	SA7	Field^∗^
Len7	0.52	0.66	0.68	0.69	0.46	0.70	0.73	0.71
Bel1		0.68	0.61	0.51	0.26	0.56	0.48	0.52
Ps31			0.78	0.66	0.45	0.74	0.72	0.75
Pr11				0.71	0.37	0.70	0.63	0.71
Vol13					0.44	0.64	0.64	0.69
G5						0.32	0.42	0.40
Can11							0.77	0.76
SA7								0.77

*^∗^Natural inoculum evaluated at the adult growth stage.*

### Adult Plant Stage Screening in the Field

In the field experiment, NFNB severity on the susceptible cultivar Carlsberg reached 50–60% leaf area infected at the time of assessment. The IR of the susceptible parent Harrington was also high (7.5 on 1–9 scale). As expected, the IR of the resistant parent CLS was low (3.0) (Supplementary Table 1).

When evaluated at the adult plant stage in the field, the CLS/Harrington DH population displayed a bi-modal distribution of resistance to *Ptt* (Figure 1). Unlike results from the seedling assays, segregation of resistance did not fit a single gene model, but instead fitted to a 1:3 (R:S) ratio, suggesting a two complementary gene inheritance model (Table 2). Interestingly, only one DH line displayed a lower IR than the resistant parent CLS. High correlations (0.71–0.77) between adult plant reaction in the field and seedling reaction were observed for five isolates: Len7, Ps31, Pr11, Can11, and SA7 (Table 3). The lowest correlation between adult plant reaction and seedling reaction was found for the isolate from Germany (G5, 0.40).

### Genomic Regions Associated With Resistance

**FIGURE 3:**
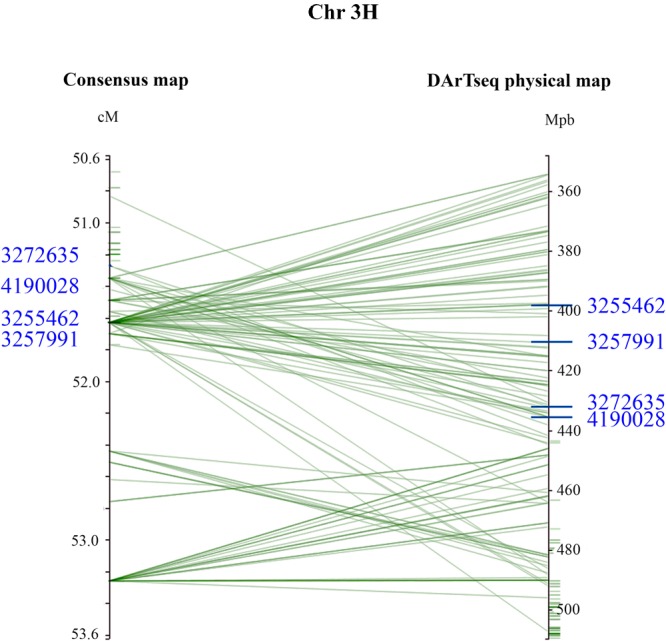
Genomic interval containing associated DArTseq markers on Chromosome 3H. The DArTseq consensus map was aligned into the barley DArTseq physical map. For visual purposes, marker interval spanning the 50.6–53.6 cM was projected. The four DArTseq markers (*blue*) displayed on the chromosome, designated as *qPttCLS*, were consistently detected in all eight assays and were present in both the consensus and physical maps. The four markers defined an interval region spanning 398203862–435526243 bp onto the physical map.

The mean IR for ‘resistant’ and ‘susceptible’ classes was clearly differentiated across the six disease assays (Table 4). Comparative MFA identified 251 DArTseq markers associated with resistance to all isolates, except G5. These markers span a region of 36.26–76.56 cM on chromosome 3H of the barley consensus genetic map (Supplementary Table 2). Marker 4016922 (44.74 cM) was identified to be the most significant (*P* = 1.80E-18) and had the highest discriminant value (D = 0.74), followed by 3270940 (50.50 cM; *P* = 2.40E-16, D = 0.61) which was associated in seedling response to isolates Vol13 and PS31, respectively. In the field, marker 3268587 was the most significant (*P* = 2.30E-17) and the highest discriminant value (D = 0.67). Alleles contributing resistance were donated by resistant parent CLS. Five DArT-seq markers were consistently detected in eight disease assays, positioned within a smaller window of 0.5 cM ranging from 51.27 to 51.77 cM (Figure 3 and Table 5). We designated this QTL region *qPttCLS*.

**Table 4:** Summary of resistant (R) and susceptible (S) classes used for Marker Frequency Analysis for the six *Pyrenophora teres* f. *teres* assays.

Assay	Class	*N*	Mean IR	*SD*
Field^∗^	R	40	4.5	1.5
	S	40	8.2	0.7
Pr11	R	40	3.8	1.4
	S	40	8.5	0.7
Ps31	R	40	3.3	0.9
	S	40	7.2	0.6
Bel1	R	40	4.2	1.2
	S	40	7.9	0.6
Len7	R	40	2.3	1.3
	S	40	7.1	0.7
Vol13	R	40	1.8	0.9
	S	40	7.0	0.7
G5	R	40	2.7	0.9
	S	40	6.4	0.3
Can11	R	40	4.4	1.7
	S	40	8.7	0.7
SA7	R	40	3.6	2.2
	S	40	8.4	0.6

*^∗^Natural inoculum evaluated at the adult growth stage.*

**Table 5:** Genetic position and discriminant values for the subset of DArT-seq markers that were consistently detected in eight marker frequency analyses performed in this study.

CloneID	Allele sequence	Genetic position (cM)	Field^∗^	Seedling						
				Pr11	Ps31	Bel31	Len7	Vol13	Can11	SA7
3272635	TGCAGGTTATACCCTCTTCTTAGGCTCCCGTGGCTT TGACTCAGCCTTCTTGATGCCGCCCTTGGCGGC	51.27	0.66	0.54	0.57	0.44	0.50	0.67	0.46	0.42
4190028	TGCAGATCGATCACCAAAAGATCCGTAGAACCGCCA ACGCCGCAAACGAGCATACCGTGAAGTGGACGG	51.35	0.66	0.50	0.58	0.41	0.49	0.62	0.44	0.42
3255462	TGCAGAGATCCTTATATCTGATTTTGGTTTCTTAGA GAGGATTTCTCCTGAATCTCTTGGTCGGATTGA	51.63	0.66	0.51	0.57	0.41	0.47	0.67	0.46	0.45
4793115	TGCAGCGGAGCTGGGGACGGCGGCGTGATCCGAGAT CGGAAGAGCGGTTCAGCAGGAATGCCGAGACCG	51.63	0.63	0.45	0.55	0.40	0.48	0.62	0.44	0.43
3257991	TGCAGCACCTCATCGACCTTTCCTCCTCTCCGTCCC TGCACCCTCTGTGAGCAGCGCAGCCCCGCCTCC	51.77	0.62	0.48	0.54	0.41	0.47	0.70	0.46	0.45

*All five markers were significantly associated with resistance to *P. teres* on Chromosome 3 (*P* < 0.001) and mark the 0.5 cM region containing *qPttCLS* donated by Canadian Lake Shore.*

*^∗^Natural inoculum evaluated at the adult growth stage.*

From these five markers, four (3255462, 3257991, 3272635, and 4190028) were positioned onto the barley physical map spanning an interval region between 398203862 and 435526175 bp. This region was found to harbor 179 genes (Supplementary Table 3) of which 27 genes with annotated functions are involved in plant disease response, cell death, and signaling pathways (Table 6).

**Table 6:** List of 27 genes defined by the four (4) DArTseq markers (*qPttCLS*) positioned onto the barley physical map on the interval region 398203862–435526243 bp.

Gene ID	Description	Function
HORVU3Hr1G053990	–	Defense response, cell death, ethylene biosynthetic process, leaf senescence
HORVU3Hr1G054090	–	Transferase activity
HORVU3Hr1G054120	–	Gene silencing by RNA
HORVU3Hr1G055130	Cytochrome c oxidase subunit 3	Aerobic electron transport chain
HORVU3Hr1G055260	–	DNA binding
HORVU3Hr1G055330	–	Lipid metabolic process
HORVU3Hr1G055410	–	Protein transport, positive regulation of signaling
HORVU3Hr1G055450	–	Integral component of membrane
HORVU3Hr1G055550	–	Protein coding
HORVU3Hr1G055620	–	Protein kinase activity
HORVU3Hr1G055630	–	Motor activity in plasma membrane
HORVU3Hr1G055650	–	Protein coding
HORVU3Hr1G055700	Uroporphyrinogen decarboxylase	Uroporphyrinogen decarboxylase activity; lyase activity
HORVU3Hr1G055870	–	Protein coding
HORVU3Hr1G055900	–	Response to dessication
HORVU3Hr1G055920	–	Protein coding
HORVU3Hr1G055990	Beta-adaptin-like protein	Intercellular or vesicle-mediated protein transport
HORVU3Hr1G056200	Mitogen-activated protein kinase	MAPK cascade; protein phosphorylation
HORVU3Hr1G056440	Pectinesterase	Cell wall modification
HORVU3Hr1G056560	–	Flavonoid biosynthetic process; oxidation-reduction process
HORVU3Hr1G056990	Carbonic anhydrase	Carbon utilization
HORVU3Hr1G057010	CASP-like protein	Integral component of membrane
HORVU3Hr1G057090	Carbonic anhydrase	Carbon utilization
HORVU3Hr1G057630	Auxin efflux carrier component	Auxin-activated signaling pathway
HORVU3Hr1G057660	Mitogen-activated protein kinase	MAPK cascade; protein phosphorylation
HORVU3Hr1G057690	–	Monolayer-surrounded lipid storage body; integral component of membrane
HORVU3Hr1G057840	–	Zinc-ion binding protein

*The corresponding description and functions are based on the database search on *EnsemblPlants* using the barley genome assembly *Hordeum vulgare* (IBSC_v2) of the International Barley Genome Sequencing Consortium. Only the genes at the start and end position of marker interval, and genes with annotated description were searched for biological, cellular, and molecular functions.*

## Discussion

In this study we identified a major genomic region (*qPttCLS*) conferring resistance to *Ptt* in the international barley differential CLS. Although segregation for resistance was consistent with a single gene when the DH population was evaluated at the seedling stage using some isolates, this model did not fit all seedling datasets and segregation observed at the adult stage in the field suggested a two complimentary gene model. Thus, while we have identified a key genomic region conferring resistance to *Ptt* in CLS, stable expression could be more complex and may involve additional genetic factors, particularly at the adult growth stage.

The high degree of variation of *Ptt* is highlighted in numerous studies using both virulence markers (Tekauz, 1990; Steffenson and Webster, 1992; Afanasenko et al., 2009; Jalli, 2010) and molecular markers (Peever and Milgroom, 1994; Rau et al., 2003; Serenius, 2006). CLS is one of nine genotypes included in the international set of barley differentials, which is used to characterize populations and virulence phenotypes of *Ptt* (Afanasenko et al., 2009). This core set of genotypes is essential to track changes in the pathogen population in the different barley growing regions around the world. A major objective is to identify the loci underpinning resistance in all differential genotypes. This insight will identify differentials that carry identical genes or haplotypes conferring resistance, and results from pathogen surveys can then be linked directly to characterized resistance genes in the host.

Mode and Schaller (1958) first reported CLS to carry resistance to *Ptt* and based on their inheritance studies, CLS appeared to contribute two major resistance genes (*Pt2* and *Pt3*). However, more recent studies examining CLS in populations derived from crosses to susceptible cultivars Pirkka and Nadja, observed segregation patterns consistent with a single gene conferring resistance (unpublished data). Although results from genetic analyses based on segregation patterns were variable in this study, the comparative MFA performed for each assay, identified a single major genomic region on Chromosome 3H. Further, correlations between all disease assays were high, except in G5. This seems to point toward variability in levels of resistance conferred by the *qPttCLS* locus. This may explain the distorted segregation pattern observed for isolates Bel1, Pr11, Can11, and SA7; and the ineffectiveness of *qPttCLS* against isolate G5. After all, segregation analyses are based on a threshold applied to ‘resistance’ based on the observed IRs. This could also be the case for the adult assessment in the field, where a two complementary gene model was found to be significant based on Chi-squared analysis. While this suggests an additional gene may be involved, variation in expression of *qPttCLS* could be influenced by environmental cues in the field, which could lead to low numbers of DH lines actually displaying high levels of resistance. Another plausible reason for this variation in IR could be the presence of isolate-specific minor resistance genes in CLS. A number of previous mapping studies have reported isolate specific QTL (Ho et al., 1996; Grewal et al., 2012; König et al., 2014; Afanasenko et al., 2015).

Several studies have detected QTL for resistance to *Ptt* on Chromosome 3H (Graner et al., 1996; Steffenson et al., 1996; Richter et al., 1998; Cakir et al., 2003; Raman et al., 2003; Yun et al., 2005; Manninen et al., 2006; Grewal et al., 2008; Gupta et al., 2010; Tenhola-Roininen et al., 2011; König et al., 2013, 2014; Afanasenko et al., 2015). Notably, the *qPttCLS* region of interest spans 51.27–51.77 cM on 3H and QTL previously mapped to this chromosome can be compared using the consensus map provided by Aghnoum et al. (2010). For instance, Yun et al. (2005) reported *Rpt-3H-4* on the short arm of chromosome 3H (57.0–66.6 cM) via analysis of the OUH602/Harrington RIL population. Steffenson et al. (1996) reported a QTL in the Steptoe/Morex population (28.7–36.6 cM on 3H), which overlaps with the *QTLUHs-3H-2* region conferring seedling resistance to NFNB (34.0–38.0 cM) identified in detached leaf tests (König et al., 2014). Further, a QTL contributing adult plant resistance, *QTLUH-3H* (45–51 cM), was reported in the DH population Uschi/HHOR3073 (König et al., 2013). While these previously reported QTL are mapped in close proximity and in some cases overlapping with the region containing *qPttCLS*, allelism testing is required to precisely determine if the resistance gene is unique or common to these other sources. This future work will involve crossing CLS to these other sources carrying resistance genes mapped to 3H and testing for segregation of resistance in the progeny.

Aligning the DArTseq markers to the barley physical position allowed further analysis of annotated genes. The *qPttCLS* region harbored genes that are involved in important plant biological, cellular, and molecular functions such as plant disease response, cell death, and signaling pathways. Interestingly, there are still many genes in this region that are uncharacterized, and thus further characterization is important that could potentially identify novel genes. The DArT-seq markers reported in this study will be useful for MAS targeting *qPttCLS* to develop barley cultivars resistant to NFNB.

## Data Availability

The raw data supporting the conclusions of this manuscript will be made available by the authors, without undue reservation, to any qualified researcher.

## Author Contributions

ED and LZ analyzed the datasets and wrote the manuscript. LH coordinated data analyses and revised the manuscript. RF revised the data and contributed to manuscript writing. AA, OB, and NL performed the disease screens, DNA extraction, and lab work required for this study. OA designed the experiments and contributed to writing of the manuscript.

## Conflict of Interest Statement

The authors declare that the research was conducted in the absence of any commercial or financial relationships that could be construed as a potential conflict of interest.

## Footnotes

1 www.diversityarrays.com

2 https://plants.ensembl.org/Hordeum_vulgare/Info/Index?db=core
