# Supplementary material for: Genetic Characterization of Resistance to Pyrenophora teres f. teres in the International Barley Differential Canadian Lake Shore
Source: Front Plant Sci. 2019 Mar 25;10:326. doi: 10.3389/fpls.2019.00326 (PMC6442539; doi:10.3389/fpls.2019.00326)
Supplement: Supplementary file 1 [file Table_1.DOCX]

***Supplementary Material***

**Genetic characterization of resistance to *Pyrenophora teres* f. *teres***

**in the international barley differential Canadian Lake Shore**

**Eric Dinglasan^1^, Lee Hickey^1*^, Laura Ziems^1^, Ryan Fowler^1^, Anna Anisimova^2^, Olga Baranova^2^, Nina Lashina^2^, Olga Afanasenko^2^**

^1^The University of Queensland, Queensland Alliance for Agriculture and Food Innovation, St Lucia, QLD 4072, Australia

^2^All Russian Institute for Plant Protection (FSBSI VIZR), St. Petersburg-Pushkin, Russia

*Correspondence:

Dr. Lee Hickey

[l.hickey@uq.edu.au](mailto:l.hickey@uq.edu.au)

**Supplementary Table 1.** Average infection type of barley DH lines Harrington / Canadian Lake Shore to *P. teres* f. *teres* isolates of different origin.

| **DH lines and parents** | **Pr11** | **Ps31** | **Bel1** | **Len7** | **Vol13** | **Can11** | **G5** | **SA7** | **Field. Russia** |
| --- | --- | --- | --- | --- | --- | --- | --- | --- | --- |
|  |  |  |  |  |  |  |  |  |  |
| **Harrington** | 8.9 | 9 | 9 | 7.4 | 8 | 10 | 7 | 7.8 | 7.5 |
| **CLS** | 3.2 | 1.5 | 1.5 | 1 | 2.4 | 2.5 | 4.4 | 2.3 | 3 |
|  |  |  |  |  |  |  |  |  |  |
| 1 | - | 3 | - | 3.3 | - | - | - | - | - |
| 2 | - | 3 | - | 5 | - | - | - | - | - |
| 3 | 6.4 | 6.7 | 6.6 | 5 | 6.3 | 6.8 | 3.8 | 7.7 | 6.5 |
| 4 | 4.5 | 4 | 3 | 5.2 | 6.3 | 5.5 | 5.2 | 7.8 | 6.5 |
| 5 | 2.8 | 3 | 3 | 1.1 | 2.3 | 4.5 | 0 | 3.5 | 3.5 |
| 6 | 8.5 | 8 | 7.4 | 8 | 7 | 7.3 | 7.5 | 7.3 | 7.5 |
| 7 | 4.5 | 3.2 | 3.3 | 7.3 | 7 | 8.7 | 2.1 | 8 | 8 |
| 8 | 4 | 4.3 | 3.3 | 2.9 | 1.3 | 3 | 5.5 | 7 | 7.5 |
| 9 | 7.6 | 7 | 8.9 | 7.5 | 7.3 | 9.3 | 7.5 | 8.5 | 7 |
| 10 | 9.1 | 4.8 | 8 | 7.5 | 7.7 | 9 | 5.3 | 7.3 | 8 |
| 11 | 6.4 | 5.6 | 3 | 4.8 | 8 | 7.3 | 4.3 | 7.8 | 9 |
| 12 | 7.8 | - | 8 | 7 | 7 | 10 | 5.2 | 8.5 | 9 |
| 13 | 7.7 | 6 | 5 | 7 | 5.8 | 8.7 | 6 | 8 | 7 |
| 14 | 6.1 | 5.3 | 5 | 4.8 | 6.5 | 7.8 | 4 | 9 | 8.5 |
| 15 | 4.4 | 4.1 | 3 | 2.3 | 2.2 | 3.5 | 5.3 | ? | 6 |
| 16 | - | - | - | 1.5 | 1.7 | 2 | 6.5 | - | 3 |
| 17 | 8.3 | 7.3 | 7.5 | 7 | 8 | 7.5 | 5.2 | 9 | 9 |
| 18 | 9.4 | 7.7 | 7.5 | 7.2 | 6.7 | 8.5 | 5.8 | 7.5 | 8 |
| 19 | 8.2 | 6.3 | 9.3 | 6 | 4 | 10 | 6 | 8.1 | 8 |
| 20 | 4.5 | 3.1 | 7.1 | 2.9 | 1.7 | 3 | 4.1 | 2 | 5 |
| 21 | 8.7 | 7.2 | 7 | 7.8 | 7.5 | 8 | 7.3 | 9 | 8.5 |
| 22 | 8.1 | 6 | 7.5 | 8 | 7 | 8 | 7.3 | 6.2 | 7.5 |
| 23 | 8 | 6 | 7.8 | 7.6 | 4.5 | 9 | 6.5 | 9.5 | 7 |
| 24 | 1.9 | 6 | - | 1.6 | 1.5 | 5.5 | 6 | 2.4 | 3 |
| 25 | 7 | 3.9 | 7.7 | 7 | 6 | 7.3 | 2.9 | 2 | 9 |
| 26 | 9.8 | 8.1 | 7.5 | 7 | 7 | 7.5 | 6.8 | 9 | 8 |
| 27 | 7.5 | 4.5 | 4.5 | 4.3 | 7 | 8.7 | 7 | 8 | 7 |
| 28 | 7.4 | 6.5 | 6.5 | 7.6 | 7 | 7.5 | 7.3 | 8.3 | 7 |
| 29 | 6.7 | 2.8 | 3.5 | 2.1 | 1 | - | 5.7 | 9 | 7 |
| 30 | 7.7 | 4.3 | 5.5 | 5.7 | 6.8 | 3 | 4.1 | 3.3 | 4.5 |
| 31 | 8.1 | 6.8 | 8.9 | 5.5 | 5 | 7.5 | 5.8 | 9 | 8 |
| 32 | 1.8 | 3.2 | 4 | 1.3 | 3.8 | 3.8 | 3.8 | 2.9 | 3.5 |
| 33 | 3 | 2.7 | - | 1.1 | 1.5 | - | 2.7 | 2 | 3 |
| 34 | 1.9 | 3 | 3.3 | 1.2 | 1.8 | 4.3 | 6.8 | - | 3 |
| 35 | 7.1 | 7.5 | 8 | 5.9 | 5.5 | 9.3 | 1.8 | 7.8 | 8 |
| 36 | 6.9 | 5 | 8.3 | 3.8 | 6.8 | 7 | 6 | 8 | 9 |
| 37 | 6.5 | 6.8 | 7.8 | 4.5 | 7 | 8 | 5.7 | 7 | 8.5 |
| 38 | 8.5 | 8.5 | 7.3 | 6.1 | 8 | 7.7 | 7 | 7.5 | 8.5 |
| 39 | 7.4 | 7.3 | 7.3 | 7.6 | 6.5 | 7 | 7.3 | 9 | 9 |
| 40 | 6 | 7.3 | 7 | 6.3 | 2.8 | 7 | 6.1 | - | 7.5 |
| 41 | 1.6 | 3.5 | 7 | 2.6 | 1.3 | 2.3 | 1.5 | 2.2 | 1.5 |
| 42 | 3 | 3 | 7.2 | 2.2 | 1.5 | 2.8 | 1.5 | 2 | 3 |
| 43 | 7.4 | 8.5 | 7.8 | 7.2 | 7.3 | 10 | 7.8 | 8 | 9 |
| 44 | 6.8 | 2.5 | 6 | 1.6 | 1.3 | 4.3 | 2.3 | 2 | 4 |
| 45 | 3 | 2.9 | 6.8 | 1.8 | 1.2 | 3 | 4 | 2.3 | 4 |
| 46 | 6.5 | 6.8 | 7.8 | 6.1 | 4.5 | 8.3 | 3 | 8.8 | 7 |
| 47 | 3.2 | 3 | 3.3 | 7 | 6.5 | 8 | 4.3 | - | 7 |
| 48 | 8.9 | 5 | 8.2 | 5.7 | 7 | 9 | 5 | 7 | 7 |
| 49 | 8.2 | 6.8 | 8.3 | 7.1 | 3.3 | 8 | 5 | 8.8 | 7.5 |
| 50 | 9.4 | 8 | 8.5 | 7 | 6.8 | 8 | 7 | 7 | 7.5 |
| 51 | 3.4 | 3.7 | 3.9 | 1.6 | 2.5 | 2.2 | 3.8 | 2.5 | 4 |
| 52 | 8.9 | 7.1 | 7.4 | 7.2 | 7.3 | 6.5 | 5.8 | 5.5 | 8 |
| 53 | 8.8 | 6.2 | 4.7 | 7.8 | 2.5 | 9 | 5.3 | 7 | 7 |
| 54 | 6.5 | 8 | 6.8 | 4.6 | 3.8 | 8.8 | 2.3 | 9.5 | 8 |
| 55 | 8.3 | 7.1 | 8.8 | 7.5 | 7 | 7 | 5.5 | 9 | 8 |
| 56 | 3.4 | 2.8 | 3.8 | 1.4 | 1 | 3.5 | 2.5 | - | 3.5 |
| 57 | 3.4 | 2.5 | 3 | 6.3 | 1 | 4.3 | 2.2 | 2.5 | 5.5 |
| 58 | 9.3 | 4.7 | 5.5 | 7 | 6.8 | 8.2 | 2.3 | 9 | 7 |
| 59 | 8.9 | 6.8 | 7.6 | 1.7 | 2.4 | 7.2 | 1.8 | 8 | 7.5 |
| 60 | 2.8 | 1.2 | 3.5 | 7.7 | 1 | 5.5 | 1.8 | 8 | 4.5 |
| 61 | 7.7 | 7.7 | 7 | 5.5 | 4.2 | 8.7 | 2 | 8.3 | 9.5 |
| 62 | 7.2 | 7 | 7.5 | 5.9 | 8 | 9 | 1.3 | 7 | 9 |
| 63 | 8 | 7.3 | 6.8 | 6.8 | 6.3 | 9 | 2.8 | 9.5 | 9.5 |
| 64 | 6.8 | 6.4 | 6.8 | 6.1 | 5 | 9 | 2.8 | 8.7 | 8.5 |
| 65 | 8.8 | 7.5 | 8.3 | 8 | 7.3 | 9 | 7.3 | 7.5 | 7 |
| 66 | 4.9 | 6 | 7.7 | 7.5 | 5.2 | 9 | 1.5 | ? | 7 |
| 67 | 8.2 | 5.6 | 8.3 | 8 | 7 | 8.7 | 3.3 | 7.5 | 7 |
| 68 | 8.6 | 7.2 | 8.5 | 7.9 | 8 | 6.8 | 1.8 | 7.8 | 7 |
| 69 | 10 | 7.1 | 8.3 | 6.1 | 7.5 | 6.8 | 6 | 7 | 8.5 |
| 70 | 2.9 | 3.7 | 3.5 | 1.4 | 1.3 | 3.8 | 4 | 1.3 | 3.5 |
| 71 | 3 | 3.5 | 7 | 1.7 | 1 | 3.3 | 2 | 2.5 | 3.5 |
| 72 | 9.2 | 6.9 | 7.4 | 6 | 4 | 5.3 | 2.3 | 2 | 8 |
| 73 | 4.1 | 2.2 | 3.7 | 1.5 | 1 | 2.8 | 1.8 | 1 | 4.5 |
| 74 | 3.5 | 3 | 4.5 | 1.1 | 1.2 | 3.5 | 3.8 | 1.5 | 6.5 |
| 75 | 3.3 | 2 | 3 | 6 | 1 | 4 | 7.5 | 4.3 | 6.5 |
| 76 | 1.8 | 2.5 | 3.5 | 1.1 | 1.5 | 2 | 4.4 | 1.8 | 3.5 |
| 77 | 7.7 | 7 | 7.8 | 7.3 | - | 8 | 7 | - | 7 |
| 78 | 6.8 | 7 | 8.2 | 7.7 | 5.5 | 7 | 5 | 8.3 | 8 |
| 79 | 6.1 | 2.3 | 4.5 | 1.2 | 4.5 | 3.2 | 1.8 | 3 | 3 |
| 80 | 9.9 | 7.5 | 8.3 | 7 | 8.5 | 9 | 7.8 | 8 | 8 |
| 81 | 3.2 | 2.2 | 4 | 1.2 | 1.7 | 1.7 | 4.5 | 2.3 | 3.5 |
| 82 | 4.5 | 2.5 | 3.3 | 1.1 | 1.5 | 2.5 | 1.3 | 3 | 4 |
| 83 | 3.1 | 3.5 | 4.4 | 1.9 | 2.3 | 4 | 7 | 3.8 | 4.5 |
| 84 | 9 | 6.3 | 6.7 | 5.5 | 3.5 | 9 | 5.2 | 9.5 | 8.5 |
| 86 | 3.2 | - | 5.6 | 1.2 | 1.2 | 5.3 | 6.8 | 1 | 4 |
| 87 | 3.5 | 6.8 | 7.3 | 5.5 | 1.2 | 10 | 5.3 | 8.5 | 6.5 |
| 88 | 8.6 | 6.2 | 7 | 4.9 | 6.5 | 10 | 2.6 | 7 | 7 |
| 89 | 2.6 | 4.2 | 3.5 | 1.9 | 1.5 | - | 4.1 | 2 | 3.5 |
| 90 | 2.5 | 3.3 | 3 | 1.5 | 2.3 | 8 | 2.7 | 1 | 3.5 |
| 91 | 4.9 | 7 | 3.3 | 1.6 | 1.3 | - | 4.8 | 5.5 | 3 |
| 92 | 9.1 | 8.5 | 3.2 | 7.7 | 4.7 | 6 | 6.9 | 8.5 | 7 |
| 92 | 9.1 | 8.5 | 3.2 | 7.7 | 4.7 | 6 | 6.9 | 8.5 | 7 |

**Supplementary Table 2.** All DArTseq markers significantly associated with resistance to *Pyrenophora teres* f. *teres* in the CLS/Harrington DH population. The discriminant values (D) are presented along with p-values (*P*) from Chi-squared analysis for each marker for each disease assay. ‘NS’ indicates the marker was not significantly associated.

| **CloneID** | **Ch** | **Genetic**  **Position (cM)** | **Field*** | | **Seedling** | | | | | | | | | | | | | |
| --- | --- | --- | --- | --- | --- | --- | --- | --- | --- | --- | --- | --- | --- | --- | --- | --- | --- | --- |
|  |  |  |  |  | **Pr11** | | **Ps31** | | **Bel1** | | **Len7** | | **Vol 13** | | **Can11** | | **SA7** | |
|  |  |  | **D** | ***P*** | **D** | ***P*** | **D** | ***P*** | **D** | ***P*** | **D** | ***P*** | **D** | ***P*** | **D** | ***P*** | **D** | ***P*** |
| 3263120 | 3 | 36.26 | NS | NS | NS | NS | NS | NS | NS | NS | NS | NS | 0.50 | 5.1E-13 | NS | NS | NS | NS |
| 3265122 | 3 | 36.76 | NS | NS | NS | NS | NS | NS | NS | NS | 0.41 | 8.2E-11 | 0.63 | 3.3E-16 | NS | NS | NS | NS |
| 3266458 | 3 | 36.76 | NS | NS | NS | NS | NS | NS | NS | NS | NS | NS | 0.51 | 4.0E-13 | NS | NS | NS | NS |
| 3276408 | 3 | 36.76 | NS | NS | NS | NS | NS | NS | NS | NS | NS | NS | 0.58 | 7.6E-15 | NS | NS | NS | NS |
| 3432159 | 3 | 36.76 | NS | NS | NS | NS | NS | NS | NS | NS | NS | NS | 0.52 | 1.5E-13 | NS | NS | NS | NS |
| 3265441 | 3 | 36.83 | NS | NS | NS | NS | NS | NS | NS | NS | NS | NS | 0.58 | 1.3E-14 | NS | NS | NS | NS |
| 3263362 | 3 | 37.11 | NS | NS | NS | NS | NS | NS | NS | NS | NS | NS | 0.50 | 5.6E-13 | NS | NS | NS | NS |
| 3267092 | 3 | 37.11 | NS | NS | NS | NS | NS | NS | NS | NS | NS | NS | 0.50 | 3.6E-13 | NS | NS | NS | NS |
| 3269909 | 3 | 37.11 | NS | NS | NS | NS | NS | NS | NS | NS | 0.44 | 1.6E-11 | 0.54 | 3.3E-14 | NS | NS | NS | NS |
| 3270107 | 3 | 37.11 | NS | NS | NS | NS | NS | NS | NS | NS | NS | NS | 0.57 | 1.1E-14 | NS | NS | NS | NS |
| 3270334 | 3 | 37.11 | NS | NS | NS | NS | NS | NS | NS | NS | NS | NS | 0.51 | 2.6E-13 | NS | NS | NS | NS |
| 3276289 | 3 | 37.11 | NS | NS | NS | NS | NS | NS | NS | NS | NS | NS | 0.54 | 5.0E-14 | NS | NS | NS | NS |
| 3397371 | 3 | 37.11 | NS | NS | NS | NS | NS | NS | NS | NS | NS | NS | 0.50 | 4.3E-13 | NS | NS | NS | NS |
| 3433742 | 3 | 37.11 | NS | NS | NS | NS | NS | NS | NS | NS | NS | NS | 0.57 | 1.1E-14 | NS | NS | NS | NS |
| 3663182 | 3 | 37.11 | NS | NS | NS | NS | NS | NS | NS | NS | NS | NS | 0.54 | 1.0E-13 | NS | NS | NS | NS |
| 3811641 | 3 | 37.11 | NS | NS | NS | NS | NS | NS | NS | NS | NS | NS | 0.53 | 1.3E-13 | NS | NS | NS | NS |
| 4187294 | 3 | 37.11 | NS | NS | NS | NS | NS | NS | NS | NS | NS | NS | 0.59 | 5.2E-15 | NS | NS | NS | NS |
| 4789441 | 3 | 37.11 | NS | NS | NS | NS | NS | NS | NS | NS | NS | NS | 0.56 | 2.3E-14 | NS | NS | NS | NS |
| 5335605 | 3 | 37.11 | NS | NS | NS | NS | NS | NS | NS | NS | NS | NS | 0.61 | 8.6E-16 | NS | NS | NS | NS |
| 3273885 | 3 | 37.25 | NS | NS | NS | NS | NS | NS | NS | NS | NS | NS | 0.42 | 2.7E-11 | NS | NS | NS | NS |
| 4186729 | 3 | 37.25 | NS | NS | NS | NS | NS | NS | NS | NS | NS | NS | 0.41 | 3.3E-11 | NS | NS | NS | NS |
| 4189898 | 3 | 37.25 | NS | NS | NS | NS | NS | NS | NS | NS | NS | NS | 0.41 | 4.0E-11 | NS | NS | NS | NS |
| 3267082 | 3 | 37.43 | NS | NS | NS | NS | NS | NS | NS | NS | NS | NS | 0.54 | 6.3E-14 | NS | NS | NS | NS |
| 3267855 | 3 | 37.43 | NS | NS | NS | NS | NS | NS | NS | NS | 0.40 | 1.7E-10 | 0.51 | 2.6E-13 | NS | NS | NS | NS |
| 3271216 | 3 | 37.43 | NS | NS | NS | NS | NS | NS | NS | NS | NS | NS | 0.54 | 6.3E-14 | NS | NS | NS | NS |
| 3271383 | 3 | 37.43 | NS | NS | NS | NS | NS | NS | NS | NS | NS | NS | 0.54 | 8.0E-14 | NS | NS | NS | NS |
| 3985246 | 3 | 37.43 | NS | NS | NS | NS | NS | NS | NS | NS | NS | NS | 0.58 | 3.4E-15 | NS | NS | NS | NS |
| 3985472 | 3 | 37.43 | NS | NS | NS | NS | NS | NS | NS | NS | 0.41 | 1.3E-10 | 0.52 | 1.8E-13 | NS | NS | NS | NS |
| 5250921 | 3 | 37.46 | NS | NS | NS | NS | NS | NS | NS | NS | NS | NS | 0.46 | 2.3E-13 | NS | NS | NS | NS |
| 3395880 | 3 | 37.5 | NS | NS | NS | NS | NS | NS | NS | NS | NS | NS | 0.51 | 3.3E-13 | NS | NS | NS | NS |
| 4196252 | 3 | 37.5 | NS | NS | NS | NS | NS | NS | NS | NS | NS | NS | 0.50 | 5.6E-13 | NS | NS | NS | NS |
| 3263758 | 3 | 37.57 | NS | NS | NS | NS | NS | NS | NS | NS | NS | NS | 0.55 | 4.2E-14 | NS | NS | NS | NS |
| 3267383 | 3 | 37.57 | NS | NS | NS | NS | NS | NS | NS | NS | NS | NS | 0.54 | 8.3E-14 | NS | NS | NS | NS |
| 3267981 | 3 | 37.57 | NS | NS | NS | NS | NS | NS | NS | NS | 0.41 | 4.7E-11 | 0.51 | 1.8E-13 | NS | NS | NS | NS |
| 3397719 | 3 | 37.57 | NS | NS | NS | NS | NS | NS | NS | NS | 0.42 | 7.5E-11 | 0.54 | 1.0E-13 | NS | NS | NS | NS |
| 3664961 | 3 | 37.57 | NS | NS | NS | NS | NS | NS | NS | NS | NS | NS | 0.57 | 1.6E-14 | NS | NS | NS | NS |
| 4194783 | 3 | 37.57 | NS | NS | NS | NS | NS | NS | NS | NS | NS | NS | 0.50 | 4.4E-13 | NS | NS | NS | NS |
| 5336712 | 3 | 37.57 | NS | NS | NS | NS | NS | NS | NS | NS | NS | NS | 0.42 | 4.5E-11 | NS | NS | NS | NS |
| 3265803 | 3 | 37.75 | NS | NS | NS | NS | NS | NS | NS | NS | NS | NS | 0.58 | 4.4E-15 | NS | NS | NS | NS |
| 3271363 | 3 | 37.75 | NS | NS | NS | NS | NS | NS | NS | NS | NS | NS | 0.61 | 1.1E-15 | NS | NS | NS | NS |
| 3259730 | 3 | 38.03 | NS | NS | NS | NS | NS | NS | NS | NS | NS | NS | 0.58 | 4.0E-15 | NS | NS | NS | NS |
| 3265108 | 3 | 38.03 | NS | NS | NS | NS | NS | NS | NS | NS | NS | NS | 0.66 | 1.2E-16 | NS | NS | NS | NS |
| 3267568 | 3 | 38.03 | NS | NS | NS | NS | NS | NS | NS | NS | NS | NS | 0.61 | 1.4E-15 | NS | NS | NS | NS |
| 3271141 | 3 | 38.03 | NS | NS | NS | NS | NS | NS | NS | NS | NS | NS | 0.49 | 1.0E-12 | NS | NS | NS | NS |
| 3271804 | 3 | 38.03 | NS | NS | NS | NS | NS | NS | NS | NS | NS | NS | 0.56 | 7.9E-15 | NS | NS | NS | NS |
| 3273310 | 3 | 38.03 | NS | NS | NS | NS | NS | NS | NS | NS | NS | NS | 0.53 | 1.0E-13 | NS | NS | NS | NS |
| 5249997 | 3 | 38.03 | NS | NS | NS | NS | NS | NS | NS | NS | NS | NS | 0.50 | 3.5E-13 | NS | NS | NS | NS |
| 5337013 | 3 | 38.03 | NS | NS | NS | NS | NS | NS | NS | NS | NS | NS | 0.41 | 5.5E-11 | NS | NS | NS | NS |
| 3265487 | 3 | 38.51 | NS | NS | NS | NS | NS | NS | NS | NS | NS | NS | 0.58 | 3.4E-15 | NS | NS | NS | NS |
| 4790282 | 3 | 38.53 | NS | NS | NS | NS | NS | NS | NS | NS | NS | NS | 0.51 | 3.3E-13 | NS | NS | NS | NS |
| 5334729 | 3 | 38.53 | NS | NS | NS | NS | NS | NS | NS | NS | NS | NS | 0.54 | 6.0E-14 | NS | NS | NS | NS |
| 3266482 | 3 | 38.56 | NS | NS | NS | NS | NS | NS | NS | NS | NS | NS | 0.65 | 1.9E-16 | NS | NS | NS | NS |
| 3926305 | 3 | 38.56 | NS | NS | NS | NS | NS | NS | NS | NS | NS | NS | 0.54 | 1.2E-13 | NS | NS | NS | NS |
| 3985992 | 3 | 38.56 | NS | NS | NS | NS | NS | NS | NS | NS | NS | NS | 0.57 | 8.9E-15 | NS | NS | NS | NS |
| 4174862 | 3 | 38.56 | NS | NS | NS | NS | NS | NS | NS | NS | NS | NS | 0.60 | 1.3E-15 | NS | NS | NS | NS |
| 5250923 | 3 | 38.56 | NS | NS | NS | NS | NS | NS | NS | NS | NS | NS | 0.57 | 8.9E-15 | NS | NS | NS | NS |
| 5336563 | 3 | 38.56 | NS | NS | NS | NS | NS | NS | NS | NS | NS | NS | 0.52 | 2.4E-13 | NS | NS | NS | NS |
| 3258528 | 3 | 39.98 | NS | NS | NS | NS | NS | NS | NS | NS | NS | NS | 0.60 | 2.6E-15 | NS | NS | NS | NS |
| 3664424 | 3 | 39.98 | NS | NS | NS | NS | NS | NS | NS | NS | NS | NS | 0.63 | 4.1E-16 | NS | NS | NS | NS |
| 3664011 | 3 | 40.65 | NS | NS | NS | NS | NS | NS | NS | NS | NS | NS | 0.69 | 1.2E-17 | NS | NS | NS | NS |
| 3664939 | 3 | 40.65 | NS | NS | NS | NS | NS | NS | NS | NS | NS | NS | 0.60 | 5.1E-16 | NS | NS | NS | NS |
| 5251533 | 3 | 40.65 | NS | NS | NS | NS | NS | NS | NS | NS | NS | NS | 0.62 | 5.7E-16 | NS | NS | NS | NS |
| 5335398 | 3 | 40.65 | NS | NS | NS | NS | NS | NS | NS | NS | NS | NS | 0.56 | 7.1E-15 | NS | NS | NS | NS |
| 3397812 | 3 | 42.07 | NS | NS | NS | NS | NS | NS | NS | NS | NS | NS | 0.56 | 9.0E-15 | NS | NS | NS | NS |
| 3264351 | 3 | 42.46 | NS | NS | NS | NS | NS | NS | NS | NS | NS | NS | 0.57 | 3.3E-15 | NS | NS | NS | NS |
| 3264995 | 3 | 42.97 | NS | NS | NS | NS | NS | NS | NS | NS | NS | NS | 0.57 | 8.9E-15 | NS | NS | NS | NS |
| 5251451 | 3 | 42.97 | NS | NS | NS | NS | NS | NS | NS | NS | NS | NS | 0.56 | 1.3E-14 | NS | NS | NS | NS |
| 3259055 | 3 | 44.26 | NS | NS | NS | NS | NS | NS | NS | NS | NS | NS | 0.59 | 7.9E-16 | NS | NS | NS | NS |
| 3263346 | 3 | 44.26 | NS | NS | NS | NS | NS | NS | NS | NS | 0.43 | 1.7E-11 | 0.66 | 5.3E-17 | NS | NS | NS | NS |
| 3267696 | 3 | 44.26 | NS | NS | NS | NS | NS | NS | NS | NS | 0.41 | 6.1E-11 | 0.67 | 6.5E-17 | NS | NS | NS | NS |
| 3271205 | 3 | 44.26 | NS | NS | NS | NS | NS | NS | NS | NS | 0.43 | 5.8E-12 | 0.68 | 1.9E-17 | NS | NS | NS | NS |
| 3925721 | 3 | 44.26 | NS | NS | NS | NS | NS | NS | NS | NS | NS | NS | 0.53 | 4.7E-14 | NS | NS | NS | NS |
| 4790821 | 3 | 44.26 | NS | NS | NS | NS | NS | NS | NS | NS | 0.41 | 6.9E-11 | 0.63 | 1.6E-16 | NS | NS | NS | NS |
| 4793572 | 3 | 44.26 | NS | NS | NS | NS | NS | NS | NS | NS | NS | NS | 0.62 | 2.8E-16 | NS | NS | NS | NS |
| 5251276 | 3 | 44.26 | NS | NS | NS | NS | NS | NS | NS | NS | NS | NS | 0.62 | 1.4E-15 | NS | NS | NS | NS |
| 4016922 | 3 | 44.74 | NS | NS | NS | NS | NS | NS | NS | NS | 0.44 | 1.5E-11 | 0.74 | 1.8E-18 | NS | NS | NS | NS |
| 3268669 | 3 | 45.15 | NS | NS | NS | NS | NS | NS | NS | NS | NS | NS | 0.52 | 2.7E-14 | NS | NS | NS | NS |
| 3396112 | 3 | 46.14 | 0.55 | 8.9E-16 | NS | NS | 0.44 | 9.9E-15 | NS | NS | 0.44 | 2.0E-13 | 0.50 | 4.4E-15 | NS | NS | NS | NS |
| 3263573 | 3 | 46.18 | 0.46 | 4.8E-12 | 0.43 | 2.7E-11 | 0.43 | 2.0E-11 | NS | NS | 0.46 | 4.7E-12 | 0.66 | 8.0E-17 | 0.46 | 1.05E-12 | NS | NS |
| 3264300 | 3 | 46.18 | 0.46 | 2.7E-12 | 0.40 | 2.4E-11 | 0.43 | 8.1E-12 | NS | NS | 0.53 | 1.7E-13 | 0.66 | 4.6E-17 | 0.46 | 2.60E-13 | NS | NS |
| 3265312 | 3 | 46.18 | 0.48 | 8.9E-13 | NS | NS | 0.41 | 1.7E-11 | NS | NS | 0.44 | 4.0E-12 | 0.60 | 1.2E-15 | 0.41 | 4.75E-12 | NS | NS |
| 3266381 | 3 | 46.18 | 0.51 | 5.2E-13 | 0.43 | 2.1E-11 | 0.43 | 1.4E-11 | 0.47 | 2.4E-12 | 0.48 | 3.7E-12 | 0.61 | 2.2E-15 | NS | NS | NS | NS |
| 3269920 | 3 | 46.18 | NS | NS | NS | NS | NS | NS | NS | NS | 0.40 | 7.3E-11 | 0.62 | 2.2E-16 | NS | NS | NS | NS |
| 3270772 | 3 | 46.18 | NS | NS | NS | NS | NS | NS | NS | NS | NS | NS | 0.59 | 1.7E-15 | NS | NS | NS | NS |
| 3272959 | 3 | 46.18 | 0.45 | 4.8E-12 | NS | NS | NS | NS | NS | NS | 0.51 | 2.9E-13 | 0.63 | 2.3E-16 | 0.46 | 4.21E-13 | 0.40 | 1.87E-11 |
| 3273065 | 3 | 46.18 | 0.49 | 7.6E-13 | 0.49 | 4.8E-13 | 0.42 | 1.1E-11 | NS | NS | 0.49 | 1.5E-12 | 0.66 | 5.0E-17 | 0.49 | 1.15E-13 | NS | NS |
| 3274128 | 3 | 46.18 | 0.48 | 1.8E-12 | 0.41 | 4.1E-11 | 0.41 | 4.9E-11 | NS | NS | 0.44 | 7.9E-12 | 0.67 | 6.1E-17 | 0.47 | 6.20E-13 | NS | NS |
| 3663060 | 3 | 46.18 | NS | NS | NS | NS | NS | NS | NS | NS | NS | NS | 0.45 | 8.4E-14 | NS | NS | NS | NS |
| 3912953 | 3 | 46.18 | 0.50 | 4.3E-13 | NS | NS | NS | NS | NS | NS | 0.43 | 2.6E-11 | 0.65 | 6.7E-17 | 0.44 | 5.24E-13 | NS | NS |
| 3986464 | 3 | 46.18 | NS | NS | NS | NS | NS | NS | NS | NS | NS | NS | 0.65 | 6.7E-17 | NS | NS | NS | NS |
| 3265570 | 3 | 46.32 | 0.58 | 4.7E-15 | 0.46 | 7.7E-13 | 0.50 | 6.1E-14 | NS | NS | 0.52 | 3.6E-13 | 0.65 | 6.6E-17 | 0.50 | 1.81E-14 | 0.52 | 2.41E-14 |
| 3666569 | 3 | 46.32 | 0.58 | 1.6E-15 | 0.50 | 1.7E-14 | 0.50 | 1.4E-14 | NS | NS | 0.53 | 5.0E-14 | 0.66 | 2.0E-17 | 0.42 | 2.54E-13 | NS | NS |
| 4013422 | 3 | 46.32 | 0.50 | 3.5E-13 | 0.41 | 1.8E-11 | 0.44 | 5.7E-12 | NS | NS | 0.43 | 1.1E-11 | 0.57 | 3.3E-15 | NS | NS | 0.43 | 2.81E-12 |
| 3274105 | 3 | 46.53 | NS | NS | NS | NS | NS | NS | NS | NS | NS | NS | 0.52 | 1.1E-13 | NS | NS | NS | NS |
| 5335523 | 3 | 46.53 | NS | NS | NS | NS | NS | NS | NS | NS | 0.43 | 1.1E-11 | 0.57 | 3.3E-15 | NS | NS | NS | NS |
| 3272299 | 3 | 46.88 | 0.45 | 6.6E-12 | NS | NS | NS | NS | NS | NS | 0.43 | 1.7E-11 | 0.63 | 2.1E-16 | 0.46 | 4.21E-13 | NS | NS |
| 5251682 | 3 | 46.88 | NS | NS | NS | NS | NS | NS | NS | NS | NS | NS | 0.49 | 1.1E-13 | NS | NS | NS | NS |
| 3433582 | 3 | 47.38 | 0.53 | 4.7E-14 | 0.44 | 2.3E-12 | 0.46 | 8.9E-13 | NS | NS | 0.42 | 1.6E-11 | 0.57 | 4.5E-15 | NS | NS | NS | NS |
| 5250288 | 3 | 48.45 | 0.56 | 6.1E-15 | 0.45 | 4.1E-13 | 0.55 | 3.5E-15 | NS | NS | 0.44 | 3.9E-12 | 0.63 | 1.3E-16 | 0.42 | 1.03E-12 | NS | NS |
| 3433399 | 3 | 48.69 | 0.56 | 7.9E-15 | 0.42 | 2.7E-12 | 0.49 | 7.5E-14 | NS | NS | 0.42 | 1.4E-11 | 0.56 | 2.4E-15 | 0.45 | 6.97E-14 | 0.42 | 6.12E-13 |
| 4015972 | 3 | 48.69 | 0.58 | 4.0E-15 | 0.43 | 2.3E-12 | 0.50 | 5.1E-14 | NS | NS | 0.46 | 3.6E-12 | 0.65 | 6.7E-17 | 0.47 | 5.30E-14 | 0.43 | 5.33E-13 |
| 3273300 | 3 | 49.29 | 0.57 | 3.5E-15 | 0.47 | 2.5E-13 | 0.49 | 9.7E-14 | NS | NS | 0.46 | 1.9E-12 | 0.61 | 2.6E-16 | NS | NS | NS | NS |
| 3917044 | 3 | 49.29 | 0.59 | 3.2E-15 | 0.41 | 1.8E-11 | 0.52 | 8.0E-14 | NS | NS | 0.44 | 6.4E-12 | 0.59 | 1.6E-15 | 0.48 | 1.45E-13 | 0.44 | 1.98E-12 |
| 5250234 | 3 | 49.29 | 0.51 | 2.1E-13 | 0.42 | 1.5E-11 | 0.45 | 3.3E-12 | NS | NS | 0.41 | 4.7E-11 | 0.59 | 1.6E-15 | 0.41 | 5.90E-12 | 0.41 | 1.91E-11 |
| 5250925 | 3 | 49.29 | 0.52 | 3.3E-14 | 0.48 | 1.0E-13 | 0.45 | 2.1E-13 | NS | NS | 0.44 | 6.5E-12 | 0.60 | 1.6E-16 | 0.42 | 1.73E-13 | NS | NS |
| 5337117 | 3 | 49.29 | 0.53 | 7.3E-14 | NS | NS | 0.41 | 4.9E-11 | NS | NS | NS | NS | 0.58 | 7.0E-15 | NS | NS | 0.49 | 6.18E-13 |
| 3396289 | 3 | 49.34 | 0.49 | 7.6E-13 | NS | NS | 0.42 | 1.1E-11 | NS | NS | 0.50 | 4.5E-13 | 0.70 | 9.9E-18 | 0.43 | 9.82E-13 | NS | NS |
| 5334390 | 3 | 50.21 | 0.49 | 1.1E-13 | NS | NS | NS | NS | NS | NS | NS | NS | 0.51 | 1.2E-14 | NS | NS | NS | NS |
| 3264067 | 3 | 50.5 | 0.57 | 1.6E-15 | 0.47 | 3.9E-14 | 0.52 | 5.4E-15 | NS | NS | 0.57 | 9.2E-15 | 0.65 | 2.7E-17 | 0.41 | 2.28E-13 | NS | NS |
| 3264867 | 3 | 50.5 | 0.57 | 1.6E-15 | 0.47 | 3.9E-14 | 0.49 | 1.4E-14 | NS | NS | 0.53 | 3.8E-14 | 0.65 | 2.7E-17 | NS | NS | NS | NS |
| 3270940 | 3 | 50.5 | 0.66 | 5.3E-17 | 0.51 | 8.9E-15 | 0.61 | 2.4E-16 | 0.41 | 1.3E-12 | 0.50 | 2.7E-13 | 0.63 | 5.1E-17 | 0.46 | 6.74E-14 | NS | NS |
| 5251174 | 3 | 50.5 | 0.56 | 7.1E-15 | 0.46 | 3.4E-13 | 0.47 | 1.9E-13 | NS | NS | 0.44 | 3.4E-12 | 0.60 | 5.6E-16 | NS | NS | NS | NS |
| 4198346 | 3 | 51.1 | 0.56 | 2.6E-15 | NS | NS | 0.49 | 1.9E-14 | NS | NS | 0.43 | 5.8E-12 | 0.55 | 3.7E-15 | NS | NS | NS | NS |
| 3265627 | 3 | 51.13 | 0.62 | 3.0E-16 | 0.43 | 5.8E-13 | 0.53 | 4.1E-15 | NS | NS | 0.50 | 1.3E-13 | 0.61 | 4.2E-16 | 0.43 | 1.35E-13 | 0.41 | 7.58E-13 |
| 3254741 | 3 | 51.2 | 0.58 | 4.6E-15 | 0.44 | 5.9E-13 | 0.50 | 7.0E-14 | NS | NS | 0.46 | 4.3E-12 | 0.67 | 1.4E-17 | 0.49 | 2.31E-14 | 0.42 | 7.31E-13 |
| 3264719 | 3 | 51.2 | 0.54 | 1.8E-14 | 0.43 | 4.4E-13 | 0.47 | 1.2E-13 | NS | NS | 0.43 | 1.2E-11 | 0.61 | 9.6E-17 | 0.42 | 1.58E-13 | NS | NS |
| 3271514 | 3 | 51.2 | 0.57 | 5.4E-15 | 0.50 | 5.7E-14 | 0.53 | 1.7E-14 | NS | NS | 0.46 | 5.6E-12 | 0.63 | 5.1E-17 | 0.44 | 1.40E-13 | 0.45 | 2.70E-13 |
| 3272635 | 3 | 51.27 | 0.66 | 5.3E-17 | 0.54 | 3.3E-15 | 0.57 | 9.1E-16 | 0.44 | 5.0E-13 | 0.50 | 2.7E-13 | 0.67 | 1.4E-17 | 0.46 | 6.26E-14 | 0.42 | 5.50E-13 |
| 3432961 | 3 | 51.27 | 0.63 | 1.8E-16 | 0.48 | 1.1E-13 | 0.55 | 2.2E-15 | NS | NS | 0.50 | 3.2E-13 | 0.59 | 8.5E-16 | 0.44 | 1.08E-13 | 0.46 | 1.74E-13 |
| 3985545 | 3 | 51.27 | 0.62 | 2.5E-16 | 0.50 | 6.6E-14 | 0.54 | 3.3E-15 | NS | NS | 0.52 | 1.7E-13 | 0.62 | 2.8E-16 | 0.46 | 7.54E-14 | 0.45 | 3.78E-13 |
| 3255171 | 3 | 51.35 | 0.57 | 8.9E-15 | 0.40 | 2.5E-11 | 0.49 | 3.4E-13 | NS | NS | 0.46 | 1.9E-12 | 0.57 | 4.8E-15 | 0.47 | 2.07E-13 | 0.50 | 2.01E-14 |
| 3266047 | 3 | 51.35 | 0.59 | 1.7E-15 | 0.40 | 7.8E-12 | 0.51 | 4.6E-14 | NS | NS | 0.48 | 3.9E-13 | 0.55 | 1.1E-14 | NS | NS | NS | NS |
| 3267537 | 3 | 51.35 | 0.62 | 2.4E-16 | 0.44 | 4.9E-13 | 0.54 | 3.0E-15 | NS | NS | 0.51 | 1.1E-13 | 0.58 | 1.3E-15 | 0.46 | 6.74E-14 | 0.42 | 6.32E-13 |
| 3398283 | 3 | 51.35 | 0.41 | 2.3E-13 | NS | NS | NS | NS | NS | NS | NS | NS | 0.49 | 5.1E-15 | NS | NS | NS | NS |
| 3986322 | 3 | 51.35 | 0.58 | 2.3E-15 | 0.48 | 1.7E-13 | 0.51 | 6.3E-14 | NS | NS | 0.47 | 1.1E-12 | 0.62 | 1.8E-16 | NS | NS | NS | NS |
| 4190028 | 3 | 51.35 | 0.66 | 5.9E-17 | 0.50 | 6.6E-14 | 0.58 | 8.3E-16 | 0.41 | 1.7E-12 | 0.49 | 6.1E-13 | 0.62 | 3.3E-16 | 0.44 | 1.40E-13 | 0.42 | 7.31E-13 |
| 4788380 | 3 | 51.35 | 0.58 | 1.2E-15 | 0.48 | 3.4E-14 | 0.54 | 3.0E-15 | NS | NS | 0.46 | 2.6E-12 | 0.62 | 7.0E-17 | 0.42 | 2.13E-13 | NS | NS |
| 3268635 | 3 | 51.42 | 0.58 | 1.2E-15 | 0.48 | 2.7E-14 | 0.53 | 3.9E-15 | NS | NS | 0.51 | 9.6E-14 | 0.62 | 7.0E-17 | NS | NS | 0.41 | 6.64E-13 |
| 4790346 | 3 | 51.42 | 0.62 | 3.7E-16 | 0.57 | 1.4E-15 | 0.57 | 1.1E-15 | NS | NS | 0.49 | 6.1E-13 | 0.60 | 1.6E-16 | 0.43 | 1.88E-13 | 0.44 | 3.78E-13 |
| 5250301 | 3 | 51.49 | 0.56 | 7.1E-15 | 0.45 | 4.8E-13 | 0.52 | 1.0E-14 | NS | NS | 0.49 | 2.7E-13 | 0.60 | 5.6E-16 | NS | NS | NS | NS |
| 5251212 | 3 | 51.49 | 0.57 | 4.8E-15 | 0.45 | 4.8E-13 | 0.49 | 1.3E-13 | NS | NS | 0.45 | 2.3E-12 | 0.60 | 3.8E-16 | NS | NS | NS | NS |
| 3272050 | 3 | 51.56 | 0.63 | 2.9E-16 | 0.45 | 1.3E-12 | 0.55 | 1.0E-14 | NS | NS | 0.48 | 7.5E-13 | 0.59 | 1.6E-15 | 0.44 | 4.70E-13 | 0.41 | 6.36E-12 |
| 4006707 | 3 | 51.56 | 0.62 | 2.5E-16 | 0.50 | 1.7E-13 | 0.51 | 3.7E-14 | NS | NS | 0.57 | 1.9E-14 | 0.51 | 5.9E-14 | 0.46 | 3.12E-13 | 0.43 | 5.35E-13 |
| 5338308 | 3 | 51.61 | 0.50 | 6.8E-14 | 0.42 | 9.9E-13 | 0.46 | 3.1E-13 | NS | NS | NS | NS | 0.53 | 5.7E-15 | NS | NS | NS | NS |
| 3255462 | 3 | 51.63 | 0.66 | 5.3E-17 | 0.51 | 9.6E-15 | 0.57 | 9.1E-16 | 0.41 | 1.3E-12 | 0.47 | 1.0E-12 | 0.67 | 1.4E-17 | 0.46 | 6.74E-14 | 0.45 | 2.43E-13 |
| 3258189 | 3 | 51.63 | 0.62 | 3.3E-16 | 0.48 | 4.0E-14 | 0.54 | 4.2E-15 | NS | NS | 0.53 | 1.4E-13 | 0.70 | 3.4E-18 | 0.45 | 9.40E-14 | 0.50 | 4.87E-14 |
| 3262826 | 3 | 51.63 | 0.66 | 5.4E-17 | 0.46 | 3.0E-13 | 0.54 | 3.3E-15 | NS | NS | 0.49 | 6.1E-13 | 0.65 | 7.9E-17 | 0.43 | 1.90E-13 | 0.51 | 4.00E-14 |
| 3263926 | 3 | 51.63 | 0.58 | 2.5E-15 | 0.48 | 1.8E-13 | 0.50 | 6.7E-14 | NS | NS | 0.47 | 1.1E-12 | 0.62 | 1.8E-16 | NS | NS | NS | NS |
| 3264098 | 3 | 51.63 | 0.57 | 3.5E-15 | 0.47 | 2.7E-13 | 0.52 | 3.3E-14 | NS | NS | 0.46 | 2.2E-12 | 0.61 | 2.6E-16 | 0.41 | 1.42E-12 | NS | NS |
| 3264592 | 3 | 51.63 | 0.54 | 3.6E-14 | 0.43 | 2.6E-12 | 0.46 | 1.0E-12 | NS | NS | 0.51 | 8.8E-14 | 0.57 | 3.3E-15 | NS | NS | NS | NS |
| 3264804 | 3 | 51.63 | 0.62 | 2.4E-16 | 0.54 | 3.1E-15 | 0.54 | 3.0E-15 | NS | NS | 0.50 | 2.7E-13 | 0.63 | 5.1E-17 | 0.43 | 1.35E-13 | NS | NS |
| 3266043 | 3 | 51.63 | 0.65 | 6.6E-17 | 0.51 | 9.6E-15 | 0.54 | 3.5E-15 | NS | NS | 0.47 | 1.5E-12 | 0.63 | 5.1E-17 | 0.43 | 1.72E-13 | NS | NS |
| 3266704 | 3 | 51.63 | 0.61 | 3.3E-16 | 0.48 | 3.1E-14 | 0.53 | 4.5E-15 | NS | NS | 0.46 | 1.7E-12 | 0.66 | 2.0E-17 | 0.47 | 3.91E-14 | 0.44 | 3.42E-13 |
| 3267536 | 3 | 51.63 | 0.62 | 3.7E-16 | 0.51 | 1.2E-14 | 0.60 | 3.5E-16 | NS | NS | 0.49 | 7.7E-13 | 0.67 | 1.4E-17 | 0.54 | 4.55E-15 | 0.47 | 1.25E-13 |
| 3268587 | 3 | 51.63 | 0.67 | 2.3E-17 | 0.44 | 2.1E-12 | 0.54 | 1.5E-14 | NS | NS | 0.47 | 1.3E-12 | 0.58 | 2.5E-15 | 0.43 | 7.06E-13 | 0.42 | 4.03E-12 |
| 3269558 | 3 | 51.63 | 0.47 | 1.5E-13 | NS | NS | NS | NS | NS | NS | 0.46 | 2.6E-13 | 0.51 | 1.2E-14 | NS | NS | NS | NS |
| 3270017 | 3 | 51.63 | 0.57 | 3.3E-15 | 0.46 | 3.4E-13 | 0.50 | 9.1E-14 | NS | NS | 0.45 | 2.3E-12 | 0.61 | 2.6E-16 | NS | NS | NS | NS |
| 3271664 | 3 | 51.63 | 0.62 | 2.5E-16 | 0.44 | 5.9E-13 | 0.55 | 2.8E-15 | NS | NS | 0.46 | 1.9E-12 | 0.65 | 6.6E-17 | 0.46 | 6.85E-14 | 0.48 | 1.03E-13 |
| 3271910 | 3 | 51.63 | 0.66 | 5.3E-17 | 0.54 | 3.3E-15 | 0.51 | 9.3E-15 | NS | NS | 0.47 | 1.0E-12 | 0.70 | 3.4E-18 | 0.46 | 6.74E-14 | NS | NS |
| 3272012 | 3 | 51.63 | 0.55 | 1.1E-14 | 0.44 | 7.0E-13 | 0.46 | 2.9E-13 | NS | NS | 0.43 | 5.0E-12 | 0.58 | 8.3E-16 | NS | NS | NS | NS |
| 3272217 | 3 | 51.63 | 0.62 | 2.4E-16 | 0.44 | 4.9E-13 | 0.54 | 3.0E-15 | NS | NS | 0.51 | 9.6E-14 | 0.62 | 3.0E-16 | 0.46 | 6.74E-14 | 0.45 | 2.43E-13 |
| 3272662 | 3 | 51.63 | 0.54 | 2.7E-14 | 0.47 | 2.1E-13 | 0.49 | 1.0E-13 | NS | NS | 0.42 | 3.0E-11 | 0.62 | 3.0E-16 | 0.47 | 1.96E-13 | 0.41 | 4.45E-12 |
| 3273058 | 3 | 51.63 | 0.57 | 3.3E-15 | 0.48 | 1.7E-13 | 0.51 | 6.3E-14 | NS | NS | 0.46 | 1.6E-12 | 0.61 | 2.6E-16 | NS | NS | NS | NS |
| 3273993 | 3 | 51.63 | 0.58 | 1.2E-15 | 0.50 | 1.2E-14 | 0.53 | 3.9E-15 | NS | NS | 0.49 | 4.5E-13 | 0.66 | 2.0E-17 | NS | NS | NS | NS |
| 3274605 | 3 | 51.63 | 0.62 | 2.8E-16 | 0.48 | 2.7E-14 | 0.54 | 3.5E-15 | NS | NS | 0.50 | 4.9E-13 | 0.63 | 5.1E-17 | 0.51 | 1.08E-14 | 0.42 | 7.32E-13 |
| 3276521 | 3 | 51.63 | 0.46 | 7.0E-14 | NS | NS | NS | NS | NS | NS | 0.44 | 2.5E-13 | 0.52 | 1.9E-15 | NS | NS | NS | NS |
| 3661983 | 3 | 51.63 | 0.60 | 2.0E-15 | 0.45 | 5.7E-13 | 0.51 | 4.5E-14 | NS | NS | 0.48 | 8.4E-13 | 0.60 | 5.7E-16 | 0.44 | 1.29E-13 | NS | NS |
| 3663565 | 3 | 51.63 | 0.58 | 2.5E-15 | 0.48 | 1.9E-13 | 0.50 | 6.7E-14 | NS | NS | 0.51 | 1.1E-13 | 0.66 | 4.5E-17 | NS | NS | 0.41 | 5.80E-12 |
| 3663721 | 3 | 51.63 | 0.59 | 9.9E-16 | 0.48 | 2.7E-14 | 0.54 | 3.5E-15 | NS | NS | 0.46 | 1.9E-12 | 0.67 | 1.4E-17 | 0.43 | 1.58E-13 | 0.48 | 9.44E-14 |
| 3664181 | 3 | 51.63 | 0.58 | 1.4E-15 | 0.48 | 3.4E-14 | 0.50 | 1.4E-14 | NS | NS | 0.50 | 1.5E-13 | 0.62 | 7.0E-17 | 0.47 | 4.20E-14 | NS | NS |
| 3913808 | 3 | 51.63 | 0.52 | 4.3E-14 | NS | NS | 0.42 | 2.9E-12 | NS | NS | 0.48 | 9.5E-13 | 0.60 | 6.8E-16 | NS | NS | NS | NS |
| 3919630 | 3 | 51.63 | 0.52 | 4.3E-14 | 0.50 | 1.6E-14 | 0.49 | 1.6E-14 | NS | NS | 0.53 | 5.0E-14 | 0.56 | 2.9E-15 | NS | NS | NS | NS |
| 3926414 | 3 | 51.63 | 0.57 | 3.3E-15 | 0.48 | 1.7E-13 | 0.50 | 9.1E-14 | NS | NS | 0.46 | 1.6E-12 | 0.61 | 2.6E-16 | NS | NS | NS | NS |
| 3926519 | 3 | 51.63 | 0.65 | 1.4E-16 | 0.46 | 1.3E-12 | 0.51 | 1.3E-13 | NS | NS | 0.45 | 3.3E-12 | 0.60 | 9.9E-16 | NS | NS | NS | NS |
| 3986534 | 3 | 51.63 | 0.59 | 1.6E-15 | 0.49 | 1.2E-13 | 0.52 | 4.4E-14 | NS | NS | 0.48 | 7.5E-13 | 0.63 | 1.3E-16 | 0.41 | 1.33E-12 | NS | NS |
| 4006735 | 3 | 51.63 | 0.59 | 9.2E-16 | 0.48 | 2.7E-14 | 0.54 | 3.2E-15 | NS | NS | 0.46 | 2.4E-12 | 0.67 | 1.4E-17 | 0.43 | 1.72E-13 | 0.44 | 3.24E-13 |
| 4006943 | 3 | 51.63 | 0.58 | 2.5E-15 | 0.48 | 1.8E-13 | 0.50 | 6.7E-14 | NS | NS | 0.47 | 1.3E-12 | 0.62 | 1.8E-16 | 0.42 | 9.36E-13 | NS | NS |
| 4195246 | 3 | 51.63 | 0.57 | 4.5E-15 | 0.45 | 4.6E-13 | 0.49 | 1.2E-13 | NS | NS | 0.46 | 1.9E-12 | 0.60 | 3.8E-16 | NS | NS | NS | NS |
| 4330203 | 3 | 51.63 | 0.59 | 1.6E-15 | 0.49 | 1.3E-13 | 0.51 | 4.7E-14 | NS | NS | 0.48 | 9.0E-13 | 0.67 | 3.2E-17 | 0.41 | 1.42E-12 | 0.40 | 8.94E-12 |
| 4790239 | 3 | 51.63 | 0.57 | 3.3E-15 | 0.47 | 2.4E-13 | 0.50 | 9.1E-14 | NS | NS | 0.46 | 1.6E-12 | 0.61 | 2.6E-16 | NS | NS | NS | NS |
| 4790731 | 3 | 51.63 | 0.55 | 1.6E-14 | NS | NS | 0.47 | 6.2E-13 | NS | NS | 0.41 | 3.2E-11 | 0.52 | 7.9E-14 | NS | NS | NS | NS |
| 4791424 | 3 | 51.63 | 0.54 | 2.7E-14 | 0.58 | 9.2E-16 | 0.52 | 2.7E-14 | NS | NS | 0.42 | 3.0E-11 | 0.67 | 1.1E-17 | 0.41 | 2.86E-13 | NS | NS |
| 4793115 | 3 | 51.63 | 0.63 | 2.9E-16 | 0.45 | 1.5E-12 | 0.55 | 1.1E-14 | 0.40 | 7.3E-12 | 0.48 | 9.0E-13 | 0.62 | 4.0E-16 | 0.44 | 5.02E-13 | 0.43 | 2.81E-12 |
| 4793569 | 3 | 51.63 | 0.51 | 1.1E-13 | 0.44 | 2.3E-12 | 0.44 | 2.3E-12 | NS | NS | 0.41 | 3.2E-11 | 0.54 | 1.1E-14 | NS | NS | NS | NS |
| 4793586 | 3 | 51.63 | 0.53 | 5.3E-14 | 0.41 | 5.4E-12 | 0.45 | 1.3E-12 | NS | NS | 0.46 | 1.9E-12 | 0.57 | 4.8E-15 | NS | NS | NS | NS |
| 5250949 | 3 | 51.63 | 0.64 | 9.3E-17 | 0.40 | 7.8E-12 | 0.50 | 6.8E-14 | NS | NS | 0.43 | 5.0E-12 | 0.55 | 1.1E-14 | NS | NS | NS | NS |
| 5251271 | 3 | 51.63 | 0.54 | 1.2E-14 | 0.49 | 1.3E-13 | 0.48 | 1.7E-13 | NS | NS | 0.43 | 5.5E-12 | 0.61 | 2.6E-16 | NS | NS | 0.41 | 1.71E-12 |
| 5251436 | 3 | 51.63 | 0.57 | 4.8E-15 | 0.47 | 2.4E-13 | 0.49 | 1.3E-13 | NS | NS | 0.50 | 1.8E-13 | 0.60 | 3.8E-16 | NS | NS | NS | NS |
| 5334896 | 3 | 51.63 | 0.47 | 4.9E-13 | NS | NS | 0.40 | 7.3E-12 | NS | NS | NS | NS | 0.51 | 4.7E-14 | NS | NS | NS | NS |
| 5336493 | 3 | 51.63 | 0.45 | 1.0E-12 | NS | NS | NS | NS | NS | NS | NS | NS | 0.49 | 9.9E-14 | NS | NS | NS | NS |
| 3265135 | 3 | 51.7 | 0.62 | 2.8E-16 | 0.51 | 9.6E-15 | 0.57 | 1.1E-15 | NS | NS | 0.50 | 3.2E-13 | 0.67 | 1.4E-17 | 0.43 | 1.46E-13 | NS | NS |
| 3269192 | 3 | 51.7 | 0.62 | 3.0E-16 | 0.54 | 3.9E-15 | 0.57 | 1.1E-15 | NS | NS | 0.53 | 1.4E-13 | 0.67 | 1.4E-17 | 0.43 | 1.88E-13 | 0.44 | 3.78E-13 |
| 3257991 | 3 | 51.77 | 0.62 | 2.2E-16 | 0.48 | 2.7E-14 | 0.54 | 3.2E-15 | 0.41 | 1.4E-12 | 0.47 | 1.2E-12 | 0.70 | 3.4E-18 | 0.46 | 7.27E-14 | 0.45 | 2.80E-13 |
| 5337015 | 3 | 51.77 | 0.45 | 1.1E-11 | NS | NS | NS | NS | NS | NS | NS | NS | 0.50 | 5.6E-13 | NS | NS | NS | NS |
| 3268840 | 3 | 52.44 | 0.62 | 2.4E-16 | 0.49 | 2.5E-14 | 0.54 | 3.0E-15 | NS | NS | 0.50 | 2.7E-13 | 0.63 | 5.1E-17 | 0.41 | 2.86E-13 | NS | NS |
| 5250874 | 3 | 52.44 | 0.54 | 3.2E-14 | 0.44 | 2.3E-12 | 0.46 | 8.9E-13 | NS | NS | 0.43 | 1.1E-11 | 0.58 | 3.1E-15 | NS | NS | NS | NS |
| 3395835 | 3 | 52.51 | 0.55 | 1.1E-14 | 0.45 | 4.8E-13 | 0.46 | 2.7E-13 | NS | NS | 0.43 | 5.0E-12 | 0.58 | 8.3E-16 | NS | NS | NS | NS |
| 4016116 | 3 | 52.51 | 0.54 | 3.2E-14 | 0.43 | 3.3E-12 | 0.46 | 8.9E-13 | NS | NS | 0.47 | 1.1E-12 | 0.58 | 3.1E-15 | NS | NS | NS | NS |
| 5338017 | 3 | 53.06 | 0.40 | 6.2E-11 | NS | NS | NS | NS | NS | NS | NS | NS | 0.44 | 8.4E-12 | NS | NS | NS | NS |
| 3263961 | 3 | 53.26 | 0.57 | 1.4E-15 | 0.48 | 2.5E-14 | 0.50 | 1.3E-14 | NS | NS | 0.50 | 1.0E-13 | 0.61 | 9.6E-17 | NS | NS | NS | NS |
| 3266150 | 3 | 53.26 | 0.61 | 8.1E-16 | 0.42 | 3.7E-12 | 0.52 | 3.1E-14 | NS | NS | 0.50 | 1.8E-13 | 0.57 | 4.8E-15 | 0.41 | 1.33E-12 | 0.42 | 1.21E-12 |
| 3267051 | 3 | 53.26 | 0.61 | 8.1E-16 | 0.47 | 2.5E-13 | 0.52 | 3.1E-14 | NS | NS | 0.46 | 1.6E-12 | 0.61 | 2.6E-16 | 0.42 | 9.36E-13 | NS | NS |
| 3268727 | 3 | 53.26 | 0.59 | 9.9E-16 | 0.49 | 2.5E-14 | 0.51 | 9.3E-15 | NS | NS | 0.47 | 1.5E-12 | 0.67 | 1.4E-17 | 0.43 | 1.46E-13 | 0.42 | 6.32E-13 |
| 3268739 | 3 | 53.26 | 0.55 | 1.2E-14 | 0.43 | 4.1E-12 | 0.44 | 5.3E-13 | NS | NS | NS | NS | 0.54 | 1.9E-14 | NS | NS | NS | NS |
| 3271967 | 3 | 53.26 | 0.59 | 1.9E-15 | 0.49 | 1.3E-13 | 0.51 | 4.7E-14 | NS | NS | 0.47 | 1.1E-12 | 0.63 | 1.3E-16 | 0.43 | 7.06E-13 | 0.42 | 4.03E-12 |
| 3396736 | 3 | 53.26 | 0.59 | 9.9E-16 | 0.49 | 2.3E-14 | 0.51 | 8.6E-15 | NS | NS | 0.47 | 1.2E-12 | 0.60 | 1.6E-16 | 0.46 | 6.26E-14 | NS | NS |
| 3665714 | 3 | 53.26 | 0.56 | 5.1E-15 | 0.46 | 3.6E-13 | 0.48 | 1.4E-13 | NS | NS | 0.45 | 2.7E-12 | 0.60 | 3.8E-16 | 0.41 | 1.33E-12 | NS | NS |
| 3666514 | 3 | 53.26 | 0.61 | 4.5E-16 | 0.50 | 1.7E-14 | 0.51 | 1.4E-14 | NS | NS | 0.48 | 1.3E-12 | 0.67 | 1.4E-17 | 0.45 | 1.28E-13 | 0.43 | 9.42E-13 |
| 3666930 | 3 | 53.26 | 0.57 | 3.3E-15 | 0.47 | 2.4E-13 | 0.50 | 9.1E-14 | NS | NS | 0.46 | 1.6E-12 | 0.61 | 2.6E-16 | NS | NS | NS | NS |
| 3985304 | 3 | 53.26 | 0.61 | 6.9E-16 | 0.48 | 2.2E-13 | 0.53 | 2.4E-14 | NS | NS | 0.47 | 1.5E-12 | 0.62 | 1.8E-16 | 0.42 | 1.15E-12 | 0.41 | 7.47E-12 |
| 5250672 | 3 | 53.26 | 0.59 | 1.7E-15 | 0.40 | 7.8E-12 | 0.50 | 6.8E-14 | 0.42 | 2.0E-13 | 0.43 | 5.0E-12 | 0.55 | 1.1E-14 | NS | NS | NS | NS |
| 5334702 | 3 | 53.26 | 0.49 | 2.4E-13 | 0.42 | 4.8E-12 | 0.42 | 4.7E-12 | NS | NS | NS | NS | 0.53 | 2.3E-14 | NS | NS | NS | NS |
| 3268129 | 3 | 54.21 | 0.57 | 4.8E-15 | 0.46 | 3.4E-13 | 0.49 | 1.3E-13 | NS | NS | 0.45 | 2.3E-12 | 0.60 | 3.8E-16 | NS | NS | NS | NS |
| 5251525 | 3 | 54.21 | 0.57 | 3.3E-15 | 0.47 | 2.4E-13 | 0.50 | 9.1E-14 | NS | NS | 0.46 | 1.6E-12 | 0.61 | 2.6E-16 | NS | NS | NS | NS |
| 4015311 | 3 | 55.1 | 0.59 | 1.8E-15 | 0.49 | 9.9E-14 | 0.48 | 1.4E-13 | NS | NS | 0.48 | 3.0E-13 | 0.60 | 5.6E-16 | NS | NS | 0.43 | 1.74E-13 |
| 3268736 | 3 | 56.44 | NS | NS | NS | NS | NS | NS | NS | NS | NS | NS | 0.42 | 2.9E-14 | NS | NS | NS | NS |
| 5257505 | 3 | 56.69 | 0.62 | 2.8E-16 | 0.48 | 2.9E-14 | 0.57 | 1.1E-15 | NS | NS | 0.47 | 1.2E-12 | 0.63 | 5.1E-17 | 0.43 | 1.72E-13 | 0.42 | 8.54E-13 |
| 3263727 | 3 | 57.08 | NS | NS | NS | NS | NS | NS | NS | NS | NS | NS | 0.42 | 2.9E-14 | NS | NS | NS | NS |
| 3433728 | 3 | 57.08 | NS | NS | NS | NS | NS | NS | NS | NS | NS | NS | 0.41 | 2.4E-13 | NS | NS | NS | NS |
| 3265861 | 3 | 57.22 | NS | NS | NS | NS | 0.42 | 1.7E-14 | NS | NS | NS | NS | 0.43 | 2.2E-14 | NS | NS | NS | NS |
| 3266741 | 3 | 57.22 | NS | NS | NS | NS | NS | NS | NS | NS | NS | NS | 0.43 | 1.3E-13 | NS | NS | NS | NS |
| 3268264 | 3 | 57.22 | 0.42 | 2.4E-13 | NS | NS | 0.42 | 1.7E-14 | NS | NS | NS | NS | 0.43 | 2.2E-14 | NS | NS | NS | NS |
| 3268936 | 3 | 57.22 | NS | NS | NS | NS | 0.42 | 1.7E-14 | NS | NS | NS | NS | 0.46 | 1.2E-14 | NS | NS | NS | NS |
| 3269462 | 3 | 57.22 | NS | NS | NS | NS | 0.42 | 1.4E-13 | NS | NS | NS | NS | 0.46 | 8.4E-14 | NS | NS | NS | NS |
| 3269900 | 3 | 57.22 | 0.42 | 2.4E-13 | NS | NS | 0.42 | 1.7E-14 | NS | NS | NS | NS | 0.41 | 3.5E-14 | NS | NS | NS | NS |
| 4189103 | 3 | 57.22 | 0.44 | 1.2E-13 | NS | NS | 0.44 | 9.9E-15 | NS | NS | NS | NS | 0.46 | 1.2E-14 | NS | NS | NS | NS |
| 4792211 | 3 | 57.22 | NS | NS | NS | NS | NS | NS | NS | NS | NS | NS | 0.42 | 1.7E-13 | NS | NS | NS | NS |
| 3269728 | 3 | 57.44 | NS | NS | NS | NS | NS | NS | NS | NS | NS | NS | 0.43 | 1.3E-13 | NS | NS | NS | NS |
| 3397510 | 3 | 57.44 | NS | NS | NS | NS | NS | NS | NS | NS | NS | NS | 0.47 | 8.7E-15 | NS | NS | NS | NS |
| 3263717 | 3 | 57.51 | NS | NS | NS | NS | NS | NS | NS | NS | NS | NS | 0.42 | 2.9E-14 | NS | NS | NS | NS |
| 3268109 | 3 | 57.51 | NS | NS | NS | NS | NS | NS | NS | NS | NS | NS | 0.41 | 2.4E-13 | NS | NS | NS | NS |
| 3276498 | 3 | 58.43 | NS | NS | NS | NS | NS | NS | NS | NS | NS | NS | 0.43 | 2.2E-14 | NS | NS | NS | NS |
| 4186721 | 3 | 58.43 | NS | NS | NS | NS | NS | NS | NS | NS | NS | NS | 0.44 | 1.7E-14 | NS | NS | NS | NS |
| 3264871 | 3 | 58.57 | NS | NS | NS | NS | NS | NS | NS | NS | NS | NS | 0.42 | 2.9E-14 | NS | NS | NS | NS |
| 3266173 | 3 | 58.75 | NS | NS | NS | NS | 0.42 | 1.7E-14 | NS | NS | 0.43 | 1.5E-12 | 0.48 | 6.3E-15 | NS | NS | NS | NS |
| 3265216 | 3 | 59.14 | NS | NS | NS | NS | NS | NS | NS | NS | NS | NS | 0.41 | 2.5E-13 | NS | NS | NS | NS |
| 3266431 | 3 | 59.42 | NS | NS | NS | NS | NS | NS | NS | NS | NS | NS | 0.46 | 1.2E-14 | NS | NS | NS | NS |
| 3267350 | 3 | 59.42 | NS | NS | NS | NS | NS | NS | NS | NS | 0.40 | 4.0E-12 | 0.51 | 2.9E-15 | NS | NS | NS | NS |
| 3270997 | 3 | 59.42 | 0.42 | 2.7E-13 | NS | NS | 0.42 | 1.7E-14 | NS | NS | NS | NS | 0.43 | 2.2E-14 | NS | NS | NS | NS |
| 3267363 | 3 | 59.56 | NS | NS | NS | NS | NS | NS | NS | NS | NS | NS | 0.46 | 6.7E-14 | NS | NS | NS | NS |
| 3271369 | 3 | 59.63 | NS | NS | NS | NS | NS | NS | NS | NS | NS | NS | 0.48 | 6.3E-15 | NS | NS | NS | NS |
| 3268386 | 3 | 60.69 | 0.47 | 6.8E-14 | NS | NS | 0.42 | 1.7E-14 | NS | NS | 0.43 | 1.8E-12 | 0.46 | 1.2E-14 | NS | NS | NS | NS |
| 4783487 | 3 | 60.84 | 0.41 | 1.6E-12 | NS | NS | 0.41 | 2.9E-13 | NS | NS | NS | NS | 0.44 | 9.5E-14 | NS | NS | NS | NS |
| 3986933 | 3 | 61.83 | NS | NS | NS | NS | 0.42 | 1.7E-14 | NS | NS | NS | NS | 0.43 | 2.2E-14 | NS | NS | NS | NS |
| 3265295 | 3 | 61.9 | NS | NS | NS | NS | NS | NS | NS | NS | NS | NS | 0.42 | 2.9E-14 | NS | NS | NS | NS |
| 3265270 | 3 | 62.68 | NS | NS | NS | NS | NS | NS | NS | NS | NS | NS | 0.42 | 9.1E-13 | NS | NS | NS | NS |
| 5250409 | 3 | 62.68 | NS | NS | NS | NS | NS | NS | NS | NS | NS | NS | 0.43 | 1.3E-13 | NS | NS | NS | NS |
| 5334417 | 3 | 62.68 | NS | NS | NS | NS | 0.49 | 2.7E-15 | NS | NS | NS | NS | NS | NS | NS | NS | NS | NS |
| 4007763 | 3 | 62.85 | NS | NS | NS | NS | NS | NS | NS | NS | NS | NS | 0.43 | 2.2E-14 | NS | NS | NS | NS |
| 3264570 | 3 | 62.96 | NS | NS | NS | NS | NS | NS | NS | NS | NS | NS | 0.41 | 3.7E-14 | NS | NS | NS | NS |
| 5334711 | 3 | 64.16 | NS | NS | NS | NS | NS | NS | NS | NS | NS | NS | 0.41 | 2.4E-13 | NS | NS | NS | NS |
| 5336771 | 3 | 67.74 | 0.47 | 1.8E-13 | NS | NS | NS | NS | NS | NS | NS | NS | 0.48 | 3.0E-14 | NS | NS | NS | NS |
| 3270873 | 3 | 67.92 | 0.41 | 3.0E-13 | NS | NS | NS | NS | NS | NS | NS | NS | NS | NS | NS | NS | NS | NS |
| 3398354 | 3 | 67.92 | NS | NS | NS | NS | NS | NS | NS | NS | NS | NS | 0.44 | 1.7E-14 | NS | NS | NS | NS |
| 5336548 | 3 | 67.99 | NS | NS | NS | NS | NS | NS | NS | NS | NS | NS | 0.46 | 1.2E-14 | NS | NS | NS | NS |
| 3273553 | 3 | 68.2 | NS | NS | NS | NS | NS | NS | NS | NS | NS | NS | 0.43 | 1.3E-13 | NS | NS | NS | NS |
| 3264526 | 3 | 69.9 | 0.40 | 4.8E-13 | NS | NS | NS | NS | NS | NS | NS | NS | 0.44 | 1.7E-14 | NS | NS | NS | NS |
| 3260204 | 3 | 73.12 | NS | NS | NS | NS | NS | NS | NS | NS | NS | NS | 0.41 | 3.7E-14 | NS | NS | NS | NS |
| 3266295 | 3 | 73.12 | 0.42 | 2.9E-14 | NS | NS | NS | NS | NS | NS | NS | NS | NS | NS | NS | NS | NS | NS |
| 3270726 | 3 | 75.71 | 0.44 | 1.7E-14 | NS | NS | NS | NS | NS | NS | NS | NS | 0.41 | 3.7E-14 | NS | NS | NS | NS |
| 3397478 | 3 | 76.56 | 0.42 | 2.9E-14 | NS | NS | NS | NS | NS | NS | NS | NS | NS | NS | NS | NS | NS | NS |

*Natural inoculum evaluated at the adult growth stage.

**Supplementary Table 3.** List of genes defined by the 4 DArTseq marker sequence on the barley physical map position spanning a region 398203862-435526243. The corresponding description and functions are based on database search on EnsemblPlants using the barley genome assembly *Hordeum vulgare* (IBSC_v2) of the International Barley Genome Sequencing Consortium. Only the genes at the start and end position, and with annotated description were searched for their biological, cellular, and molecular functions.

| **Gene ID** | **Description** | **Functions** |
| --- | --- | --- |
| HORVU3Hr1G053990 |  | defense response, cell death, ethylene biosynthetic process, leaf senescence |
| HORVU3Hr1G054030 |  |  |
| HORVU3Hr1G054050 |  |  |
| HORVU3Hr1G054070 |  |  |
| HORVU3Hr1G054090 | Predicted protein [Source:UniProtKB/TrEMBL] | transferase activity |
| HORVU3Hr1G054100 |  |  |
| HORVU3Hr1G054120 | Predicted protein [Source:UniProtKB/TrEMBL] | gene silencing by RNA |
| HORVU3Hr1G054160 |  |  |
| HORVU3Hr1G054170 |  |  |
| HORVU3Hr1G054200 |  |  |
| HORVU3Hr1G054230 |  |  |
| HORVU3Hr1G054240 |  |  |
| HORVU3Hr1G054250 |  |  |
| HORVU3Hr1G054330 |  |  |
| HORVU3Hr1G054360 |  |  |
| HORVU3Hr1G054370 |  |  |
| HORVU3Hr1G054420 |  |  |
| HORVU3Hr1G054440 |  |  |
| HORVU3Hr1G054470 |  |  |
| HORVU3Hr1G054480 |  |  |
| HORVU3Hr1G054490 |  |  |
| HORVU3Hr1G054500 |  |  |
| HORVU3Hr1G054510 |  |  |
| HORVU3Hr1G054540 |  |  |
| HORVU3Hr1G054600 |  |  |
| HORVU3Hr1G054630 |  |  |
| HORVU3Hr1G054640 |  |  |
| HORVU3Hr1G054660 |  |  |
| HORVU3Hr1G054680 |  |  |
| HORVU3Hr1G054710 |  |  |
| HORVU3Hr1G054730 |  |  |
| HORVU3Hr1G054770 |  |  |
| HORVU3Hr1G054780 |  |  |
| HORVU3Hr1G054810 |  |  |
| HORVU3Hr1G054870 |  |  |
| HORVU3Hr1G054880 |  |  |
| HORVU3Hr1G054900 |  |  |
| HORVU3Hr1G054920 |  |  |
| HORVU3Hr1G054930 |  |  |
| HORVU3Hr1G054960 |  |  |
| HORVU3Hr1G054970 |  |  |
| HORVU3Hr1G054980 |  |  |
| HORVU3Hr1G055000 |  |  |
| HORVU3Hr1G055020 |  |  |
| HORVU3Hr1G055030 |  |  |
| HORVU3Hr1G055040 |  |  |
| HORVU3Hr1G055050 |  |  |
| HORVU3Hr1G055070 |  |  |
| HORVU3Hr1G055090 |  |  |
| HORVU3Hr1G055100 |  |  |
| HORVU3Hr1G055120 |  |  |
| HORVU3Hr1G055130 | Cytochrome c oxidase subunit 3 [Source:UniProtKB/TrEMBL] |  |
| HORVU3Hr1G055160 |  |  |
| HORVU3Hr1G055170 |  |  |
| HORVU3Hr1G055180 |  |  |
| HORVU3Hr1G055200 |  |  |
| HORVU3Hr1G055260 | Predicted protein [Source:UniProtKB/TrEMBL] | DNA binding |
| HORVU3Hr1G055330 | Predicted protein [Source:UniProtKB/TrEMBL] | lipid metabolic process |
| HORVU3Hr1G055350 |  |  |
| HORVU3Hr1G055370 |  |  |
| HORVU3Hr1G055410 | Predicted protein [Source:UniProtKB/TrEMBL] | protein transport, positive regulation of signalling |
| HORVU3Hr1G055420 |  |  |
| HORVU3Hr1G055450 | Predicted protein [Source:UniProtKB/TrEMBL] | integral component of membrane |
| HORVU3Hr1G055460 |  |  |
| HORVU3Hr1G055470 |  |  |
| HORVU3Hr1G055480 |  |  |
| HORVU3Hr1G055520 |  |  |
| HORVU3Hr1G055550 | Predicted protein [Source:UniProtKB/TrEMBL] | protein coding |
| HORVU3Hr1G055570 |  |  |
| HORVU3Hr1G055600 |  |  |
| HORVU3Hr1G055620 | Predicted protein [Source:UniProtKB/TrEMBL] | protein kinase activity |
| HORVU3Hr1G055630 | Predicted protein [Source:UniProtKB/TrEMBL] | motor activity in plasma membrane |
| HORVU3Hr1G055650 | Predicted protein [Source:UniProtKB/TrEMBL] | protein coding |
| HORVU3Hr1G055670 |  |  |
| HORVU3Hr1G055700 | Uroporphyrinogen decarboxylase [Source:UniProtKB/TrEMBL] |  |
| HORVU3Hr1G055710 |  |  |
| HORVU3Hr1G055720 |  |  |
| HORVU3Hr1G055730 |  |  |
| HORVU3Hr1G055740 |  |  |
| HORVU3Hr1G055750 |  |  |
| HORVU3Hr1G055780 |  |  |
| HORVU3Hr1G055820 |  |  |
| HORVU3Hr1G055830 |  |  |
| HORVU3Hr1G055840 |  |  |
| HORVU3Hr1G055870 | Predicted protein [Source:UniProtKB/TrEMBL] | protein coding |
| HORVU3Hr1G055900 | Predicted protein [Source:UniProtKB/TrEMBL] | response to dessication |
| HORVU3Hr1G055910 |  |  |
| HORVU3Hr1G055920 | Predicted protein [Source:UniProtKB/TrEMBL] | protein coding |
| HORVU3Hr1G055930 |  |  |
| HORVU3Hr1G055940 |  |  |
| HORVU3Hr1G055950 |  |  |
| HORVU3Hr1G055960 |  |  |
| HORVU3Hr1G055970 |  |  |
| HORVU3Hr1G055980 |  |  |
| HORVU3Hr1G055990 | Beta-adaptin-like protein [Source:UniProtKB/TrEMBL] |  |
| HORVU3Hr1G056090 |  |  |
| HORVU3Hr1G056100 |  |  |
| HORVU3Hr1G056110 |  |  |
| HORVU3Hr1G056120 |  |  |
| HORVU3Hr1G056130 |  |  |
| HORVU3Hr1G056170 |  |  |
| HORVU3Hr1G056200 | Mitogen-activated protein kinase [Source:UniProtKB/TrEMBL] |  |
| HORVU3Hr1G056230 |  |  |
| HORVU3Hr1G056270 |  |  |
| HORVU3Hr1G056330 |  |  |
| HORVU3Hr1G056350 |  |  |
| HORVU3Hr1G056370 |  |  |
| HORVU3Hr1G056400 |  |  |
| HORVU3Hr1G056430 |  |  |
| HORVU3Hr1G056440 | Pectinesterase [Source:UniProtKB/TrEMBL] |  |
| HORVU3Hr1G056450 |  |  |
| HORVU3Hr1G056470 |  |  |
| HORVU3Hr1G056500 |  |  |
| HORVU3Hr1G056540 |  |  |
| HORVU3Hr1G056550 |  |  |
| HORVU3Hr1G056560 | Predicted protein [Source:UniProtKB/TrEMBL] | flavonoid biosynthetic process; oxidation-reduction process |
| HORVU3Hr1G056570 |  |  |
| HORVU3Hr1G056580 |  |  |
| HORVU3Hr1G056600 |  |  |
| HORVU3Hr1G056630 |  |  |
| HORVU3Hr1G056640 |  |  |
| HORVU3Hr1G056650 |  |  |
| HORVU3Hr1G056660 |  |  |
| HORVU3Hr1G056680 |  |  |
| HORVU3Hr1G056690 |  |  |
| HORVU3Hr1G056710 |  |  |
| HORVU3Hr1G056770 |  |  |
| HORVU3Hr1G056800 |  |  |
| HORVU3Hr1G056820 |  |  |
| HORVU3Hr1G056830 |  |  |
| HORVU3Hr1G056930 |  |  |
| HORVU3Hr1G056960 |  |  |
| HORVU3Hr1G056980 |  |  |
| HORVU3Hr1G056990 | Carbonic anhydrase [Source:UniProtKB/TrEMBL] |  |
| HORVU3Hr1G057000 |  |  |
| HORVU3Hr1G057010 | CASP-like protein [Source:UniProtKB/TrEMBL] |  |
| HORVU3Hr1G057020 |  |  |
| HORVU3Hr1G057090 | Carbonic anhydrase [Source:UniProtKB/TrEMBL] |  |
| HORVU3Hr1G057110 |  |  |
| HORVU3Hr1G057130 |  |  |
| HORVU3Hr1G057140 |  |  |
| HORVU3Hr1G057180 |  |  |
| HORVU3Hr1G057190 |  |  |
| HORVU3Hr1G057200 |  |  |
| HORVU3Hr1G057240 |  |  |
| HORVU3Hr1G057270 |  |  |
| HORVU3Hr1G057280 |  |  |
| HORVU3Hr1G057300 |  |  |
| HORVU3Hr1G057320 |  |  |
| HORVU3Hr1G057330 |  |  |
| HORVU3Hr1G057370 |  |  |
| HORVU3Hr1G057380 |  |  |
| HORVU3Hr1G057390 |  |  |
| HORVU3Hr1G057400 |  |  |
| HORVU3Hr1G057420 |  |  |
| HORVU3Hr1G057440 |  |  |
| HORVU3Hr1G057490 |  |  |
| HORVU3Hr1G057530 |  |  |
| HORVU3Hr1G057540 |  |  |
| HORVU3Hr1G057550 |  |  |
| HORVU3Hr1G057560 |  |  |
| HORVU3Hr1G057570 |  |  |
| HORVU3Hr1G057630 | Auxin efflux carrier component [Source:UniProtKB/TrEMBL] |  |
| HORVU3Hr1G057650 |  |  |
| HORVU3Hr1G057660 | Mitogen-activated protein kinase [Source:UniProtKB/TrEMBL] |  |
| HORVU3Hr1G057680 |  |  |
| HORVU3Hr1G057690 | Predicted protein [Source:UniProtKB/TrEMBL] | monolayer-surrounded lipid storage body; integral component of membrane |
| HORVU3Hr1G057700 |  |  |
| HORVU3Hr1G057710 |  |  |
| HORVU3Hr1G057720 |  |  |
| HORVU3Hr1G057730 |  |  |
| HORVU3Hr1G057740 |  |  |
| HORVU3Hr1G057750 |  |  |
| HORVU3Hr1G057760 |  |  |
| HORVU3Hr1G057770 |  |  |
| HORVU3Hr1G057780 |  |  |
| HORVU3Hr1G057810 |  |  |
| HORVU3Hr1G057820 |  |  |
| HORVU3Hr1G057840 |  | zinc-ion binding protein |

Description and function were according to EnsemblPlants (https://plants.ensembl.org/Hordeum_vulgare/Info/Index)
